# Genetic and Functional Diversity of Nitrilases in Agaricomycotina

**DOI:** 10.3390/ijms20235990

**Published:** 2019-11-28

**Authors:** Lenka Rucká, Martin Chmátal, Natalia Kulik, Lucie Petrásková, Helena Pelantová, Petr Novotný, Romana Příhodová, Miroslav Pátek, Ludmila Martínková

**Affiliations:** 1 Laboratory of Molecular Genetics of Bacteria, Institute of Microbiology of the Czech Academy of Sciences, Vídeňská 1083, CZ-142 20 Prague, Czech Republic; rucka@biomed.cas.cz; 2 Laboratory of Biotransformation, Institute of Microbiology of the Czech Academy of Sciences, Vídeňská 1083, CZ-142 20 Prague, Czech Republic; chmatal@biomed.cas.cz (M.C.); petraskova@biomed.cas.cz (L.P.); petr.novotny@biomed.cas.cz (P.N.); romana.prihodova@biomed.cas.cz (R.P.); 3 Center for Nanobiology and Structural Biology, Institute of Microbiology of the Czech Academy of Sciences, Zámek 136, CZ-373 33 Nové Hrady, Czech Republic; kulik@nh.cas.cz; 4 Laboratory of Molecular Structure Characterization, Institute of Microbiology of the Czech Academy of Sciences, Vídeňská 1083, CZ-142 20 Prague, Czech Republic; pelantova@biomed.cas.cz

**Keywords:** Basidiomycota, Agaricomycotina, nitrilase, cyanide hydratase, nitrile, substrate specificity, overproduction, homology modeling, substrate docking, phylogenetic distribution

## Abstract

Nitrilases participate in the nitrile metabolism in microbes and plants. They are widely used to produce carboxylic acids from nitriles. Nitrilases were described in bacteria, Ascomycota and plants. However, they remain unexplored in Basidiomycota. Yet more than 200 putative nitrilases are found in this division via GenBank. The majority of them occur in the subdivision Agaricomycotina. In this work, we analyzed their sequences and classified them into phylogenetic clades. Members of clade 1 (61 proteins) and 2 (25 proteins) are similar to plant nitrilases and nitrilases from Ascomycota, respectively, with sequence identities of around 50%. The searches also identified five putative cyanide hydratases (CynHs). Representatives of clade 1 and 2 (NitTv1 from *Trametes versicolor* and NitAg from *Armillaria gallica*, respectively) and a putative CynH (NitSh from *Stereum hirsutum*) were overproduced in *Escherichia coli*. The substrates of NitTv1 were fumaronitrile, 3-phenylpropionitrile, β-cyano-l-alanine and 4-cyanopyridine, and those of NitSh were hydrogen cyanide (HCN), 2-cyanopyridine, fumaronitrile and benzonitrile. NitAg only exhibited activities for HCN and fumaronitrile. The substrate specificities of these nitrilases were largely in accordance with substrate docking in their homology models. The phylogenetic distribution of each type of nitrilase was determined for the first time.

## 1. Introduction

Nitrilases (NLases; EC 3.5.5.-) constitute branch 1 of the nitrilase (NLase) superfamily, which comprises 13 branches of hydrolases acting on non-peptide C-N bonds [1]. NLases have attracted the attention of academia and industry due to their ability to hydrolyze nitriles under mild conditions and with stereo- or regioselectivities [2,3,4]. They are also promising for bioremediation, especially as far as their cyanide-degrading subtypes cyanide hydratases (CynHs; EC 4.2.1.66) and cyanide dihydratases (CynDs; EC 3.5.5.1) are concerned [5]. Our understanding of the natural roles of NLases and the cyanide-degrading enzymes still falls behind our knowledge of their biotechnological applications. Nevertheless, some probable functions of these enzymes were proposed such as nitrogen recycling, protection against herbivores, participation in the metabolism of cyano glycosides and glucosinolates, or the detoxification of hydrogen cyanide (HCN) [6].

The first NLases characterized were almost exclusively of bacterial origin [7], with some exceptions of fungal NLases, which, however, were not sequenced [8,9,10]. The bacterial NLases were usually classified into a number of substrate-specificity subtypes, i.e., aromatic NLases, aliphatic NLases, arylacetoNLases, bromoxynil-specific NLases, CynHs and CynDs [7]. However, a recent study suggested that aliphatic NLases may not actually exist, since the activities for aliphatic nitriles are present in other NLase types such as arylacetoNLases [11]. The plant NLases could be classified into two main subtypes, namely NIT1 through NIT3 with broad substrate specificities on the one hand [12] and NIT4 with a strict specificity for β-cyanoalanine (β-CA) on the other [13].

Recently, more than 10 new NLases in fungi of the division Ascomycota were characterized [14,15,16]. These enzymes could be roughly classified into aromatic NLases with a preference for (hetero) aromatic nitriles and arylacetoNLases with a preference for phenylacetonitrile (PAN) or mandelonitrile (MN). In contrast, NLases in the second largest division of fungi, Basidiomycota, have been underexplored. A single NLase was characterized in this taxon (in *Auricularia subglabra*, formerly *Auricularia delicata*) [16]. Yet understanding the function of the nitrile-converting enzymes in this taxon seems to be important with regard to the interactions of these fungi with plants. For instance, pathogenic fungi are among the factors causing damage to agricultural crops. Understanding the defense mechanisms of these fungi can lead to the identification of new targets for their suppression. In contrast, biocontrol fungi as components of the plant microbiome are known to exhibit beneficial effects on plants [17].

Basidiomycota comprise saprophytic and parasitic fungi equipped with a wealth of enzymes enabling them to degrade and utilize the plant biomass. Thus the occurrence of peroxidases, laccases, cellulases and hemicellulases in these fungi has been well documented (e.g., [18,19]). Nitrile-degrading enzymes, if active in these fungi, may support their growth on plant biomass by detoxifying natural nitriles, which are widespread in the plant kingdom [20]. For instance, toxic β-CA is formed by β-CA synthase from cysteine and HCN, whereas NIT4 transforms β-CA into a mixture of asparagine (Asn), aspartic acid (Asp) and ammonia. Thus this pathway not only serves for the detoxification of β-CA but also for nitrogen recycling. HCN, which is produced in plants during the synthesis of ethylene (phytohormone) from methionine or by cyano glycoside breakdown, can be also removed directly through its hydration catalyzed by CynHs in fungi [5].

Studies describing the transformation of nitriles or HCN in Basidiomycota are rare. Thus a psychrophilic “basidiomycete” synthesized 2-aminopropionitrile or 4-amino-4-cyanobutyric acid from HCN, ammonia and the corresponding aldehyde. The hydrolysis of these nitriles proceeded in the same organism, producing alanine and glutamic acid, respectively [21,22]. However, the enzymes catalyzing the synthesis or hydrolysis of these nitriles were not identified and the organism was not classified taxonomically. Another study reported on the elimination of CN^−^ in some species of Basidiomycota (*Trametes versicolor*, *Phanerochaete chrysosporium*, *Pleurotus sajor-caju*) but the mechanism of this process was not explained and no degradation products were detected except for a small amount of ammonia [23].

This work focused on the NLases in Agaricomycotina (mushroom-forming fungi). The aim of this work is to analyze their sequences, which are retrieved from the GenBank, and to predict their enzyme activities from sequence similarities to characterized NLases and from homology models. The sequences are classified into clades and representatives of three clades overproduced in *Escherichia coli* for the first time. The phylogenetic distribution of the homologues of the experimentally confirmed NLases is determined.

## 2. Results

### 2.1. Occurrence of Putative Nitrilases (NLases) in Agaricomycotina

The GenBank database was searched for putative NLases in Basidiomycota (subdivisions Agaricomycotina, Ustilaginomycotina and Pucciniomycotina). The templates were the bacterial NLase from *Rhodococcus rhodochrous* (GenBank: ABO46008.1) [24], plant NLases NIT1 (GenBank: NP_851011.1) [12] and NIT4 (GenBank: AAM65906) [13] from *Arabidopsis thaliana*, fungal NLases from *Fusarium verticillioides* (formerly *Gibberella moniliformis*; GenBank: ABF83489.1) [15] and *Auricularia subglabra* (formerly *Auricularia delicata*; EJD42068.1) [16] and a CynH from *Aspergillus niger* (GenBank: ABX75546) [25]. The sequences which exhibited at least 25% identity (>60% cover) to at least one of the templates were retrieved. These sequences were approximately 200 in total, and the majority of them were in Agaricomycotina (around 150 sequences). If sequences exhibiting identities of ≥ 99% to each other occurred in the same species, one of them was discarded, as well as the sequences with a length atypical of NLase (≤300 or ≥400 amino acids).

**Figure 1:**
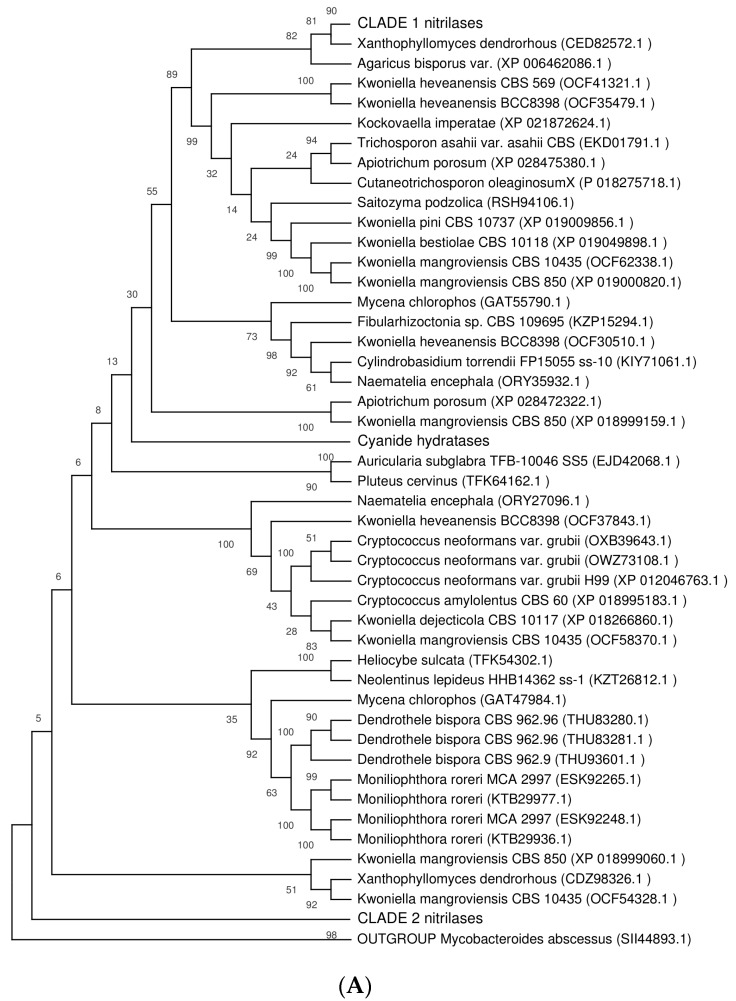

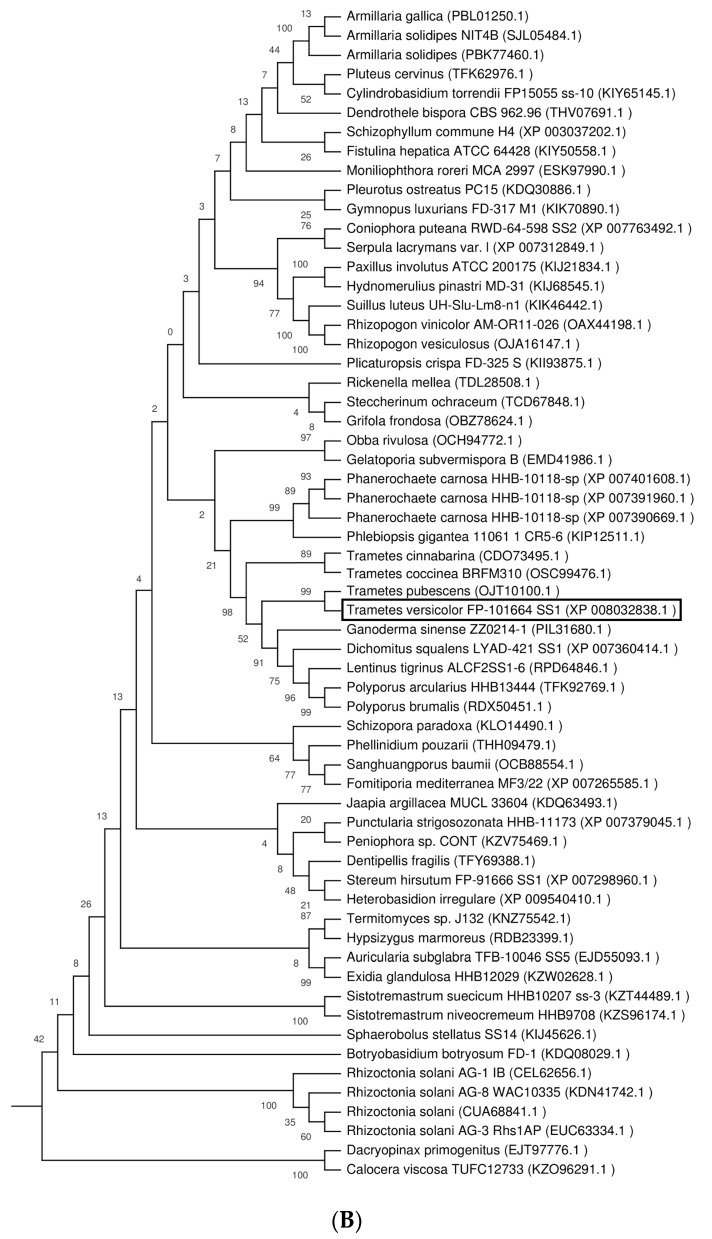

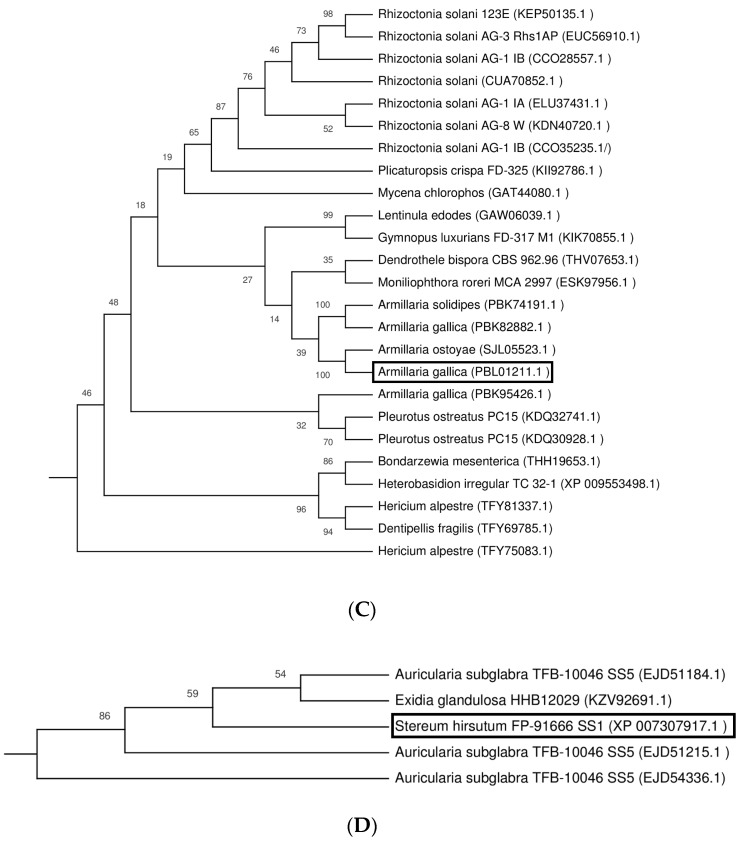
Phylogeny of nitrilases. (**A**) Full tree, (**B**) clade 1, (**C**) clade 2, (**D**) cyanide hydratases. The most parsimonious trees are shown. The consistency index is (0.340025), the retention index was (0.706964), and the composite index was 0.242788 (0.240385) for all sites and parsimony-informative sites (in parentheses). The percentage of replicate trees, in which the associated taxa clustered together in the bootstrap test (500 replicates) is shown above the branches [26]. Zero values above branches correspond to bifurcating branches and appeared as a result of tree rooting. The sequence from bacteria *Mycobacteroides abscessus* (SII44893.1) was used as the outgroup. The full tree was obtained using the subtree-pruning-regrafting (SPR) algorithm [27] with search level 1 in which the initial trees were obtained by the random addition of sequences (10 replicates). This analysis involved 135 amino acid sequences. The nitrilases (NLases) selected for overproduction are in frame.

Deleting the highly similar sequences and the sequences with atypical lengths resulted in obtaining a set of 135 sequences in Agaricomycotina (Supplementary Materials, Table S1). This set was used to construct a phylogenetic tree (Figure 1A) which enabled the identification of a few clades. The largest of them (clade 1; Figure 1B) consisted of 61 sequences similar to plant NLases (NIT1, NIT4) with identities of around 50%; nitrilases outside clade 1 showed lower identities to plant NLases. A smaller clade (clade 2; Figure 1C) was identified, consisting of 25 sequences with maximum ca. 50% identity to characterized NLases in Ascomycota as their closest homologues. The third clade (Figure 1D) only contained five sequences with identities of over 60% to the characterized CynH from *A. niger*. Therefore these proteins could be predicted as CynHs with a high probability and the clade was designated accordingly. Proteins outside the three clades could form an outgroup.

### 2.2. Selection of Nitrilases for Overproduction, Multiple Alignment and Substrate Specificity Predictions

Three of the hypothetical proteins were selected for heterologous production in *E. coli*. Two of them represented the major clades of hypothetical NLases found in Basidiomycota. In addition, one of the putative CynHs was selected. These proteins originated from *Trametes versicolor* (protein NitTv1; GenBank: XP_008032838.1; clade 1), *Armillaria gallica* (protein NitAg; PBL01211.1; clade 2) and *Stereum hirsutum* (NitSh; GenBank: XP_007307917.1; CynH). The identity levels between the three proteins were below 30%.

**Figure 2:**
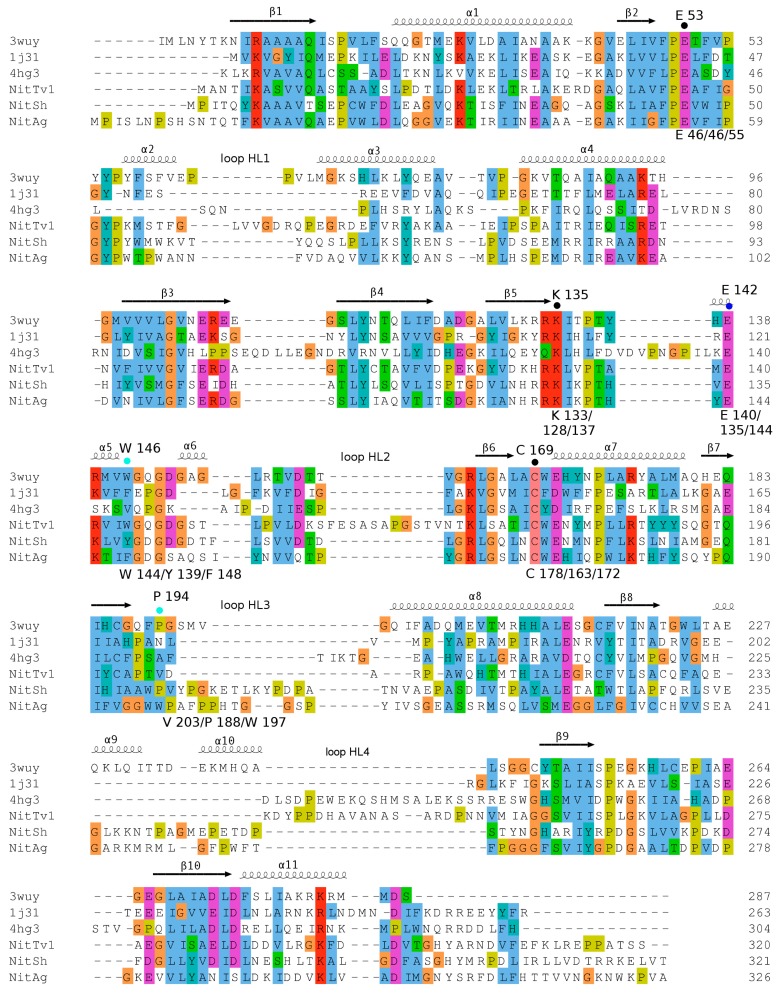
Multiple sequence alignment used for homology modeling. Parts of C-termini in NitAg and NitSh (compare Figure S1) were cut due to a missing template. Secondary structure elements are assigned based on 3wuy. The catalytic triad (E, K, C) is labeled with black dots, an additional E residue important for the catalytic mechanism [7] is marked with a blue dot. Other aa residues which are close to the catalytic center and could influence substrate recognition are marked with cyan dots. The numbers of aa residues above the alignment correspond to 3wuy, and those below the alignment to NitTv1/NitSh/NitAg NLases. Loops which are different in 3wuy and fungal NLases are labeled (HL1-HL4).

BLAST searches in the pdb database were carried out in order to identify suitable templates that enabled us to construct three-dimensional models of the selected NLases. The search revealed that all these NLases had the highest identities (30–32%) to the NLase from *Synechocystis* sp. (3wuy) [28]. In addition, NitTv1 had a 26% identity to an uncharacterized NLase-related protein 1j31 from branch 13 of the NLase superfamily (a putative apolipoprotein N-acyltransferase from *Pyrococcus horikoshii*) and to the bacterial thermophilic NLase 3ivz [29]. The 3ivz protein exhibits a high-sequence identity (82%) to 1j31. Furthermore, the structural identity of the proteins is also high, as 75.15% of the heavy atoms of both structures can be superimposed with total distance below 1Å as calculated by Yasara GDT analysis. Therefore, 3ivz does not add any new information to fungal nitrilase sequence description and it was not used in the final sequence alignment. Lower identities were found between yeast ω-amidase designated Nit2 (4hg3) [30] and NitAg (23%) or between the pyrimidine-degrading enzyme 2vhh (EC: 3.5.1.6, branch 5) and NitSh (22%). Protein 2vhh is missing many parts of the sequence (not shown); therefore, it was discarded from further analysis. A multiple sequence alignment of the fungal NLase sequences with pdb structures was constructed (Figure 2).

Previously, aligning NLases with proteins from other branches of the NLase superfamily demonstrated typical insertions in NLases [31]. Generally, the fungal NLases studied here have longer insertions (loops) than the NLase-related protein 1j31 but also NLase 3wuy. NitTv1 has longer loops, namely HL1, HL2 and HL4, while NitSh and NitAg have a longer HL3 (Figure 2). Loop HL4 in 3wuy was designated “substrate binding” and it was proposed that the larger size of this loop was the reason for the broader substrate range of the NLase from *Synechocystis* sp. in comparison with the thermophilic NLase from *Pyrococcus abyssi* [28]. This loop is similar in 3wuy and NitAg but longer in NitTv1 and NitSh.

**Figure 3:**
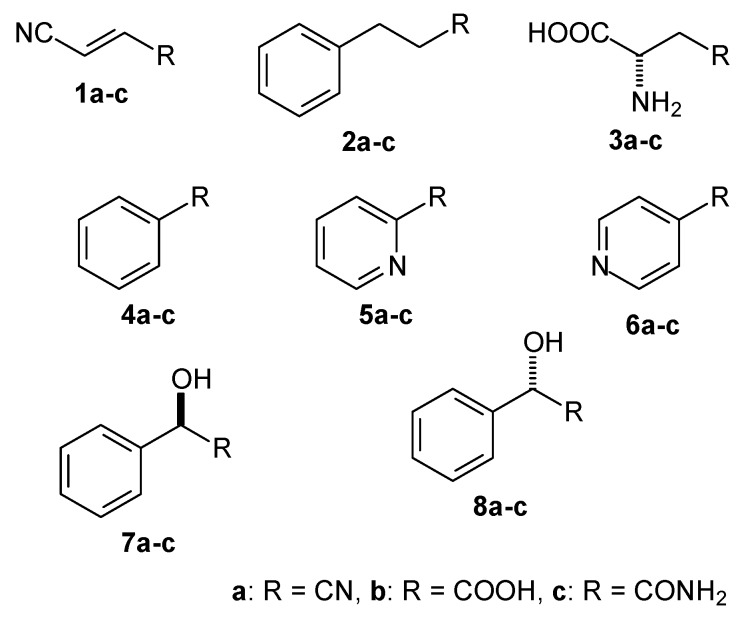
Nitriles examined as potential substrates of nitrilases NitTv1, NitAg and NitSh: fumaronitrile (**1a**), 3-phenylpropionitrile (**2a**), β-cyano-L-alanine (**3a**), benzonitrile (**4a**), 2-cyanopyridine (**5a**), 4-cyanopyridine (**6a**), (*R*)-mandelonitrile (**7a**), (*S*)-mandelonitrile (**8a**) and potential products of their biotransformations (carboxylic acids **1b**–**8b**, amides **1c**–**8c**).

The model of NitTv1 was built using three templates: 3wuy, 1j31 (HL3 loop) and 4hg3 (HL4 loop). The other two models were based on 3wuy alone. A few typical substrates of NLases and CynHs (**1a**–**8a** in Figure 3 and HCN) were docked in the models.

The substrate binding in the catalytic center of the enzyme was characterized by the binding (Glide) score. Lower values of this score correspond to better binding. However, this parameter may not be sufficient to characterize the binding of small substrates such as nitriles, in which the binding score is higher due to a lack of hydrogen-bonding (HB) or stacking interactions. Thus the binding scores may be higher with nitriles than with other (larger) substrates but must not be positive. Additional geometrical constraints were also applied. Their selection was based on the analysis of the yeast ω-amidase Nit2 (4hg3)-oxaloacetate (reaction product) complex (4hg5) [30], and on the NLase catalytic mechanism [32] in which the catalytic E forms a HB with the cyano nitrogen. This N-atom must be positioned within HB distance of the catalytic K, and also close to the catalytic E [32,33]. Furthermore, the correct orientation of the substrate was characterized by the distance of the C-atom in the CN group from the S-atom in the catalytic C (optimally < 3.1 Å). The broadest substrate specificity was predicted for NitSh. In its model, the Glide scores suggested its possible activities for fumaronitrile (FN) (**1a**), β-CA (**3a**), benzonitrile (BN) (**4a**), 2-cyanopyridine (2CP) (**5a**), (*R*)-MN (**7a**), (*S*)-MN (**8a**) and HCN. The distance between the C-atom of the substrate and the S-atom in the catalytic C indicated a suitable orientation of these substrates, being below or only slightly above 3.1 Å. The model of NitTv1 exhibited suitable Glide scores and substrate orientation with FN, 3-phenylpropionitrile (PPN) (**2a**) and β-CA. In NitAg, these docking parameters were only suitable with FN and HCN (Table 1).

### 2.3. Modeling Substrate Interaction with the Active Site Residues

**Figure 4:**
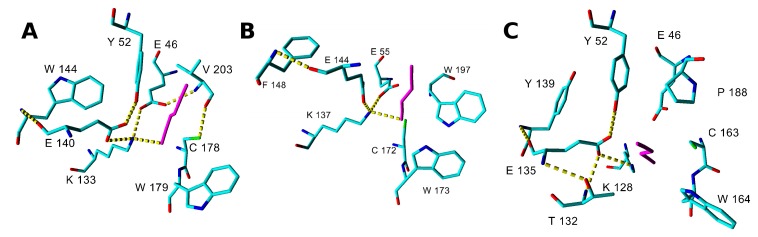
Snapshots of the complexes of nitrilase (**A**) NitTv1, (**B**) NitAg and (**C**) NitSh with fumaronitrile after 5 ns of molecular dynamics simulations. Hydrogen bonds (HBs) are shown with yellow dashed lines, fumaronitrile is colored magenta, protein residues have element color, and hydrogens are hidden. Amino acids within 3 Å from ligand and those marked with dots in Figure 2 are shown; some residues are hidden for clarity.

**Figure 5:**
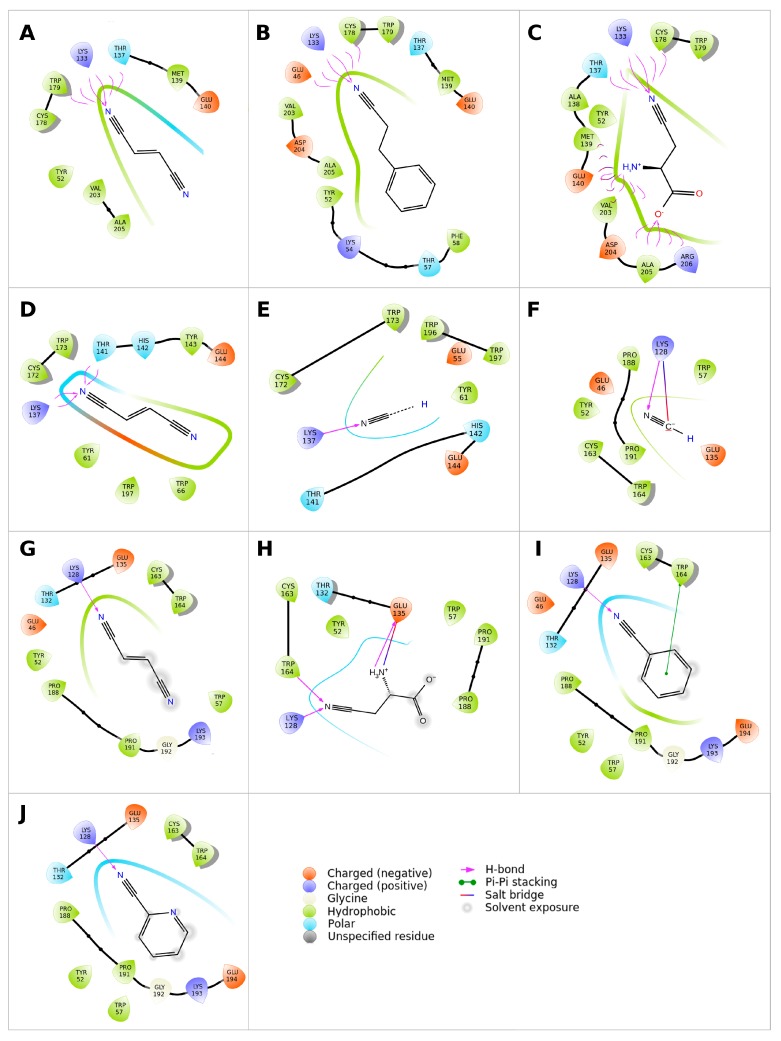
Orientation of ligands docked in the active site of nitrilases NitTv1 (**A**–**C**), NitAg (**D**,**E**) and NitSh (**F**–**J**). Enzyme residues within 3.9 Å from ligands are shown as raindrops and colored according to their properties (see legend in the right bottom angle). Ligands are represented as stick model; carbons and hydrogens are hidden: (**A**,**D**,**G**) fumaronitrile; (**B**) 3-phenylpropionitrile; (**C**,**H**) β-cyano-l-alanine; (**E**,**F**) HCN; (**I**) benzonitrile; (**J**) 2-cyanopyridine.

The catalytic residues E, K, C interact by HB and the second E residue participates in Lys stabilization by a salt bridge (Figure 4 and Supplementary materials, Table S2). Another residue found in a close contact with the ligand corresponds to W146 in 3wuy (W144, F148 and Y139 in NitTv1, NitAg and NitSh, respectively; Figure 4, Figure 5 and Figure S2).

The corresponding Y142 in the NLase from *Rhodococcus rhodochrous* was proposed to be important for the substrate specificity of the enzyme: the replacement of an aromatic residue with a non-aromatic one (A, V or L) abolished the enzyme activity for aliphatic nitriles [33]. All fungal NLases examined here have an aromatic residue at this position except for the yeast Nit 2 protein (pdb code 4hg3). This residue is not directly in the active site, but close to the catalytic E (E140, E144 and E135 in NitTv1, NitAg and NitSh, respectively; Figure 4) and it may stabilize its correct orientation through a HB formed by its backbone atoms. The exact mechanism of discrimination of substrate affinity by aromaticity of this residue is not clear. However, mutation to a smaller A residue may allow more space for catalytic E side chain rotation.

Another residue which could influence the substrate recognition corresponds to P194 in 3wuy (V203, W197 and P188 in NitTv1, NitAg and NitSh, respectively; Figure 4 and Figure 5). P194 is not conserved in NLases. In 3wuy it was identified as one of the hydrophobic residues in the “substrate-binding loop” [28]. W197 together with F200 in NitAg narrows the binding site, suggesting that the enzyme will not accept big substrates such as PPN, in contrast to NitTv1 (Supplementary materials, Figure S2). Most of the sequences in clade 2 (similar to NitAg) have a W residue in the corresponding position. From other clades only KII93875.1 from *Plicaturopsis crispa* (assigned to clade 1) has W in this position, while other clade 1 sequences have mostly a nonpolar (hydrophobic) V, A or P substitution. Clade 2 sequences share conserved WWP (residues 196–198 in NitAg) motif and loop HL3. NitSh has WPV residues in the corresponding position. Hence we could conclude that a mutation at this point could modulate the substrate affinity: smaller residues should allow bigger substrates.

The residue one position downstream of the catalytic C (W179, W173 and W164 in NitTv1, NitAg and NitSh, respectively) is also close to the substrate (Figure 4 and Figure 5). The majority of nitrilases in Ascomycota also have a W residue at this position [34] like 3wuy. The aromatic residue at this position seems to be important for the charge stabilization upon catalysis and participates in T-shaped stacking with aromatic substrates (Figure 5I). However there are some sequences in the dataset (see Supplementary materials, Table S1), which have a CGE instead of the CWE motif, e.g., in KIY71061.1 (*Cylindrobasidium torrendii*), KZP 15294.1 (*Fibularhizoctonia* sp.), OCF30510.1 (*Kwoniella heveanensis*), ORY35932.1 (*Naematelia encephala*), or other residues after catalytic C, e.g., in ELU37431.1 (*Rhizoctonia solani*), XP_012046763.1, OXB39643.1 and OWZ73108.1 (*Cryptococcus neoformans*), XP_007401608.1 (*Phanerochaete carnosa*) and THH19653.1 (*Bondarzewia mesenterica*). The sequence from *P. carnosa* belonged to clade 1 and those from *R. solani* and *B. mesenterica* to clade 2 but the other ones were not classified into any of these clades.

### 2.4. Determination of Nitrilase Activities in *E. coli* Cells

Isopropyl β-D-1-thiogalactopyranoside (IPTG) induced whole cells were examined for their activities towards typical NLase and CynH substrates which were also used for docking (see above). Preliminary screening of the substrates was carried out using high-performance liquid chromatography (HPLC) or spectrophotometric methods suitable for determining the expected products (see Materials and Methods). Thus the reaction mixtures from the transformations of substrates **1a**–**6a** (Figure 3) and HCN were checked for the presence of the corresponding acids or amides by HPLC. The retention times and ultraviolet/visible (UV/vis) spectra of the products were compared with those of authentic compounds **1b**–**6b**, **1c**, **3c**, **5c** and **6c** (commercial standards except for **1b** and **1c**, which were prepared in our previous work) [16]. The reaction mixtures from the transformations of HCN were examined for the production of formamide, which was determined spectrophotometrically and by HPLC. The production of ammonia from β-CA was determined spectrophotometrically.

The reaction products were detected for FN, PPN, 4CP and β-CA in NitTv1, for FN, BN, 2CP and HCN in NitSh, and for FN and HCN in NitAg. No products or only traces of products were determined in other cases. The identity of the products was then confirmed by NMR (acids **1b**, **3b**; amides **1c**, **2c**) and/or LC-MS (acids **1b**, **2b**, **5b**, **6b**; amides **3c**, **5c**, **6c**) (Supplementary materials, Figures S3–S13). Benzoic acid (**4b**) was identified by comparing its retention time and UV spectrum to those of the authentic standard (λmax 228.7 nm).

The major products were largely carboxylic acids but amides were produced in significant amounts in some cases. Thus amide (formamide) was produced as the major product from HCN by NitSh and NitAg. NitTv1 transformed β-CA (**3a**) into a mixture of Asp (**3b**) and Asn (**3c**) at a ratio of 72:28. Significant amounts of amides **5c** and **6c** were also found in the products from 2CP (**5a**) and 4CP (**6a**) obtained with NitSh and NitTv1, respectively. The transformation of FN (**1a**) largely proceeded on only one of the cyano groups. The ratio of acid **1b** and amide **1c** depended on the origin of the enzyme. NitTv1 produced more cyano amide **1c** than cyano acid **1b** in contrast to the other two enzymes. The products of NitAg were only detected with high cell densities (OD_600_ of ca. 10). NitTv1 also formed a minor product **1d** (Figure S3) and traces of fumaric acid (not shown) as a result of the transformation of both cyano groups in FN.

The specific activities for the aforementioned substrates were quantified from the rates of product formation determined by HPLC or spectrophotometry (Table 2). FN was the preferred substrate of NitTv1, while β-CA, PPN and 4CP were transformed at much lower rates (< 8%). The most favored substrate of NitSh was HCN, followed by 2CP, FN and BN transformed at rates one to two orders of magnitude lower. NitAg also exhibited its highest activity for HCN but this activity was almost 50 times lower than at NitSh.

The experimentally determined activities were in accordance with the predictions, however with some exceptions. NitSh exhibited the broadest substrate specificity (for HCN, 2CP, FN and BN) in accordance with the docking experiments. Nevertheless, the activity predicted for β-CA was not confirmed in this enzyme. The preference of NitTv1 for β-CA, FN, PPN and 4CP and NitAg for HCN corroborated the predictions. However, a significant discrepancy between the docking experiments and the activity assays was observed with MN (the former indicated its suitable orientation in the active sites of NitSh and NitTv1 but no activity was determined in the latter). With NitSh, this may be explained by the racemization of MN resulting in the formation of HCN and benzaldehyde, followed by the removal of HCN by this enzyme. Therefore, MN cannot be restored and benzaldehyde remains in the reaction mixture.

### 2.5. Phylogenetic Distribution of NLases in Basidiomycota

In the subdivision Agaricomycotina, NLase sequences were found in 18 orders and 64 genera (Table 3). The order with the highest number of NLases (38) was Agaricales, followed by Tremellales and Polyporales with 21 and 16 NLase sequences, respectively. The highest numbers of NLase sequences were found in the genera *Kwoniella* (13), *Rhizoctonia* (11), *Armillaria* (8) and *Moniliophthora* (6).

Sequences of clade 1 were found in 13 orders, while those of clade 2 only occurred in four orders. Four orders contained sequences of both clades. Sequences of clade 1 were most frequent in the orders Agaricales and Polyporales with 14 and 16 sequences, respectively. These sequences occurred in the classes Agaricomycetes and Dacrymycetes but not in the class Tremelomycetes (orders Tremellales and Cystofilobasidiales). Clade 2 NLases were only present in the classes Agaricomycetes and they most often occurred in the orders Agaricales (13) followed by Cantharelasses (seven; all of them in genus *Rhizoctonia*) and Russulases (five). CynHs were also only found in *Agaricomycetes*, and they were quite rare, only occurring in three genera, i.e., *Auricularia*, *Exidia* (Auriculariales) and *Stereum* (Russulales). Sequences not classified into the aforementioned clades were mainly found in Tremellales (21) and Agaricales (10).

## 3. Discussion

Basidiomycota are comprised of almost 30,000 known species with 70% of them in their largest subdivision, Agaricomycotina, which, in turn, has Agaricomycetes as its largest class (the smaller ones are Dacrymycetes and Tremellomycetes, together comprising only ca. 2% of the species [35]). In accordance with the sizes of these classes, the searches for putative NLases in Basidiomycota revealed that the majority of these enzymes occurred in the class Agaricomycetes. The study focused on the analysis of a set of 135 non-redundant putative NLases from Agaricomycotina, aiming to elucidate the function and taxonomical distribution of this type of protein.

In this NLase set, a few phylogenetic clades could be distinguished, first of all the two largest clades with 61 and 25 sequences. The distribution of the clades seemed to be taxon-specific. According to the current status of the database, clade 1 occurred in Agaricomycetes and Dacrymycetes, while clade 2 was only present in Agaricomycetes. Putative CynHs were also only found in this class. Tremellomycetes contained a few putative NLases, but these proteins did not belong to any of the aforementioned clades.

To shed light on the activities and functions of the putative fungal NLases from Agaricomycotina, two approaches were used–modeling and substrate docking on one hand and heterologous gene expression followed by activity assays on the other. The results of both approaches were in good accordance with only a few exceptions.

These strategies enabled us, with some degree of certainty, to assign the substrate specificities to the largest NLase clades in Agaricomycotina. The closest characterized homologue of NitTv1 (representative of clade 1) was the NLase NIT4 from *Arabidopsis thaliana* (GenBank: AAM65906) with 52% identity (98% cover), but NIT1, NIT2 and NIT3 NLase isoforms from the same organism (GenBank: NP_001078234.1, CAA68934.3 and NP_190018.1, respectively) also shared relatively high identities (47–49%, 98–99% cover) to NitTv1. NIT1-NIT3 on one hand, and NIT4 on the other have distinct substrate specificities: NIT4 was found to have β-CA (an intermediate of cyanide assimilation [6]) as its preferential substrate and hydrolyzed PPN (product of glucosinolate metabolism in plants [6]) with activities more than 100 times lower than those for β-CA [13]. In contrast, NIT1-3 operated on different substrates, primarily arylaliphatic nitriles such as PPN, whereas the conversion of β-CA was less than 1% compared to PPN [12]. Due to these differences between NIT1-NIT3 and NIT4, it was not possible to predict the substrate specificity of NitTv1 on the basis of sequence similarities. Whole-cell assays suggested that NitTv1 was active for both β-CA and PPN (in accordance with the docking experiments) unlike both types of plant NLases. In addition, NitTv1 transformed FN, a substrate not examined with NIT4 [13] or NIT1-NIT3 [12], with activities ca. 12.5 times higher compared to β-CA.

The similarity of NLases from clade 1 to plant NLases suggests that Basidiomycota could obtain the corresponding genes from plants by horizontal gene transfer (HGT). NIT4 is ubiquitous in plants, in contrast to NIT1-3, which is typical for Brassicaceae [12]. It may be hypothesized that the clade 1 NLases in Basidiomycota evolved from the widespread NIT4 enzymes and acquired new substrate specificities and functions. The NLases of clade 1 are the most widespread within Basidiomycota. They have been most frequently found in Agaricales and Polyporales. The transfer of the genes between plants and Basidiomycota is plausible due to frequent interactions between these organisms. For instance, many members of the order Agaricales are symbionts, saprophytes or pathogens of plants, and Polyporales are abundant in wood-rotting species (saprophytes but also pathogens). *Rhizoctonia solani* (Cantharellales), a species with the highest number of NitTv1 homologues, is a plant pathogen able to infect a number of various hosts such as rice, corn, sorghum, bean, soybean, cabbage, lettuce and many others [36].

The closest characterized homologues of NitAg, which was selected as a representative of clade 2, are various NLases from Ascomycota [15,16] with maximum ca. 50% identity to NitAg. Of the tested substrates, only HCN and FN were transformed by this enzyme. The major reaction product of HCN was formamide.

The NitAg homologues are much less abundant in Basidiomycota than NitTv1 homologues and mainly occur in a few genera of the orders Agaricales and Russulales, and in *Rhizoctonia solani* (Cantharellales). In particular *R. solani* is rich in NitAg homologues, which may be due to the exposure of the pathogen to cyanides produced by the plant host. The hypothesis of HGT may be also applied to the NLases in clade 2. It is possible that Basidiomycota acquired these genes from Ascomycota, which co-infect the same host. In fact the infection of a plant host by multiple pathogenic species is common [37].

Similarly, HGT is possible with CynHs, which rarely occur in Basidiomycota but are frequent in Ascomycota, in which a number of CynHs originating from, e.g., *Gloeocercospora sorghi*, *Neurospora crassa*, *Gibberella zeae* or *Aspergillus nidulans* were expressed in heterologous hosts such as *Aspergillus nidulans* or *E. coli* [38,39]. Furthermore, sequenced genomes of Ascomycota contain numerous putative CynHs. In contrast, only five CynHs were identified in Basidiomycota so far (in Auriculariales and Russulales, i.e., saprophytes but also plant pathogens such as *S. hirsutum*). In this case, the prediction of these enzymes as CynHs was possible due to highly conserved primary sequences (with identities of over 60% to characterized CynHs). This assumption was confirmed by activity assays with NitSh from *S. hirsutum*. Its substrate specificity for FN and 2CP, in addition to HCN, was similar to those of some previously characterized CynHs from e.g., *A. niger*, *Botryotinia fuckeliana* or *Pyrenophora teres* [16,25]. NitSh was most similar to the CynH from *N. crassa* (84.5% identity, 94% cover), which was partly characterized previously and was found to be the most stable CynH of those tested [39]. Therefore, the utility of NitSh in the bioremediation of cyanide-containing wastes will be examined.

FN was a substrate of all three of the examined enzymes. This is in accordance with our previous finding that FN was hydrolyzed by all tested NLases of different types and origin [16]. However, the products were dependent on the enzyme. NitTv1 mainly produced the corresponding cyano amide like the previously examined NLase from *Aspergillus kawachii*, which was classified as an “aromatic NLase” [16]. The major product of FN transformation by NitSh was cyano acid instead. This reaction pattern was similar to that of the NLase NitMp from *Macrophomina phaseolina* [16].

In summary, the genomes of Basidiomycota contain genes encoding functional NLases, with the majority of them occurring in the subdivision Agaricomycotina, class Agaricomycetes. The NLases in Agaricomycotina were classified into clades with different substrate specificities and different taxonomical distributions. Three of the enzymes were for the first time characterized in terms of their substrate specificities. Two of them (from *T. versicolor* and *S. hirsutum*) were similar to NLases from other organisms, namely NLases from plants and CynHs from Ascomycota. The third enzyme (from *A. gallica*) had no close characterized homologues among NLases from other organisms. NLases in Basidiomycota need to be studied further with focus on the regulation of their production in fungi. This should help to better understand and exploit the bioremediation potential of these fungi.

## 4. Materials and Methods

### 4.1. Sequence Analysis

The GenBank database was searched for NLase sequences using the program BLAST (Available online: http://blast.ncbi.nlm.nih.gov/Blast.cgi) with published NLase sequences (see section Results) as templates. For phylogenetic analysis, multiple sequence alignment was built with the MUSCLE algorithm [40]. Non-informative sites were deleted, i.e., the N- and C-terminal parts were truncated and the regions with less than 90% site coverage were eliminated during alignment preparation in MEGA X [41]. There were a total of 282 positions in the final dataset. Evolutionary analyses were conducted in MEGA X and used for phylogenetic tree building with the maximum parsimony method (500 bootstraps). The cut-off parameter used to isolate the clades from the global tree was a bootstrap value of ≥ 90%. The taxonomical classification of the strains in which NLases were sequenced was according to available online: https://www.ncbi.nlm.nih.gov/Taxonomy.

### 4.2. Nitrilase Modeling and Substrate Docking

BLAST was used to search for homologous structures in the Protein Data Bank (pdb) database [42]. Multiple sequence alignments were carried out using the program T-Coffee [43] and manually corrected in Jalview [44] based on structure alignment with Yasara [45]. Sequences with the highest identities to the examined NLases were selected as templates for homology modeling with MODELLER 9.16 [46]. The observed models were validated with the structure validation tool MOLPROBITY [47] and minimized using a water model of TIP3P type in Yasara. Structures of substrates were downloaded from the Pubchem database (Available online: https://pubchem.ncbi.nlm.nih.gov/) and minimized with the LigPrep tool in the software Schrödinder [48]. The docking of potential substrates was done using the program Glide (Schrödinger, LLC).

### 4.3. Nitrilase Overproduction

The synthesis of the NLase genes, which were optimized for *E. coli*, was performed with GeneArt (ThermoFisher Scientific, Regensburg, Germany). The optimized gene sequences are shown in Supplementary Material—Gene and protein sequences (Figure S1). Gene cloning and strain cultivation were as described previously [16]. Briefly, the expression vector was constructed by cloning the gene into the *NdeI* and *XhoI* sites of pET22b(+) and used to transform *E. coli* Origami B (DE3) co-expressing the GroEL/ES chaperone. The cultures were grown in 2 × YT medium and the induction of NLase and chaperone gene expression was performed with 0.02 mM IPTG and 0.011 mM L-arabinose, respectively, at 20 °C for 24 h. The cells were centrifuged and used for enzyme assays.

### 4.4. Nitrilase Activity Assays

The assays were carried out as described previously [16] with minor modifications. Eppendorf tubes (1.5-mL) contained 0.5 mL of the reaction mixture consisting of 50 mM Tris/HCl buffer, pH 8.0, 150 mM NaCl, 25 mM substrate, 5% (*v*/*v*) methanol (cosolvent) and an appropriate amount of whole cells. The substrate was BN, PPN, FN or MN. The reactions with 2CP, 4CP, β-CA or free cyanide (KCN) as substrates were carried out analogously but without methanol. The reactions proceeded at 30 °C with shaking, and were terminated after 5–10 min by the addition of 0.050 mL of 2 M HCl for nitriles except β-CA. The reaction of β-CA was terminated by centrifugation of the cells. The reaction of HCN was terminated by adding 1.0 mL of methanol and centrifugation. The activities for β-CA were calculated from the production of ammonia, which was determined spectrophotometrically [11,49]. The activities for HCN were determined from the production of formamide, which was determined spectrophotometrically [50] and by HPLC (see below). The activities for other substrates were determined from the production of carboxylic acids and/or amides, which were determined by HPLC (see below).

### 4.5. Analytical High-Performance Liquid Chromatography (HPLC)

The concentrations of PPN, BN and MN, and their reaction products were determined using a Chromolith SpeedRod RP-18 column (50 mm × 4.6 mm; Merck KgaA, Darmstadt, Germany) and acetonitrile (ACN)/water (20/80, *v*/*v*) with 0.1% phosphoric acid as mobile phase at a flow rate of 2 mL/min. The concentrations of FN, 2CP and 4CP, and their reaction products were determined using an ACE C8 column (5 µm, 250 mm × 4 mm; Advanced Chromatography Technologies Ltd., Aberdeen, U.K.) with ACN/5 mM sodium phosphate buffer, pH 7.2 (10/90, *v*/*v*) at a flow rate of 0.9 mL/min. Formamide was determined by hydrophilic interaction HPLC [51] under modified conditions using a Nucleosil silica column (5 µm, 250 mm × 4.6 mm; Hichrom, Lutterworth, U.K.) with ACN/water (90/10, *v*/*v*) at a flow rate of 1 mL/min. The column temperature was 34 °C in all methods. The retention times and UV spectra of the substrates and products were in accordance with those of the authentic standards.

### 4.6. Liquid Chromatography–Mass Spectrometry (LC–MS) Analysis

The reaction mixtures were analyzed using a Shimadzu Prominence system consisting of a DGU-20A_3_ mobile phase degasser, two LC-20AD solvent delivery units, a SIL-20AC cooling autosampler, a CTO-10AS column oven, an SPD-M20A diode array detector and a liquid chromatography–mass spectrometry (LC–MS-2020) mass detector with a single quadrupole equipped with an electrospray ion source (Shimadzu Europa GmbH, Duisburg, Germany). The column was an ACE 5 C8 (5 µm, 250 mm × 4 mm; Advanced Chromatography Technologies Ltd., Aberdeen, U.K.) or Chromolith RP 18e (100 mm × 3 mm; Merck KgaA, Darmstadt, Germany) and the mobile phase consisted of ACN/water (10/90). Analysis was performed at a flow rate of 0.4 mL/min and a column temperature of 34 °C. The MS parameters were as follows: negative mode; ESI interface voltage, 4.5 kV; detector voltage, 1.15 kV; nebulizing gas flow, 1.5 mL/min; drying gas flow, 15 mL/min; heat block temperature, 200 °C; DL temperature, 250 °C; SCAN mode 50–250 *m*/*z*; software LabSolutions version 5.75 SP2.

### 4.7. Nuclear Magnetic Resonance (NMR) Analysis

Nuclear magnetic resonance (NMR) analysis was performed on Bruker AVANCE III 400 and 700 MHz spectrometers (Bruker BioSpin, Rheinstetten, Germany) in deuterated dimethylsulfoxide (DMSO-*d*_6_) or D_2_O at 30 °C. Residual signal of solvents (DMSO-*d*_6_: *δ*_H_ 2.499 ppm, *δ*_C_ 39.46 ppm; D_2_O: *δ*_H_ 4.508 ppm) served as internal standards; the carbon spectra in D_2_O were referenced to the acetone signal (*δ*_C_ 30.50). NMR experiments were performed using standard manufacturers’ software.

## Figures and Tables

**Table 1:** Docking of selected substrates in fungal NLase models.

Proposed Substrate	Docking Glide Score [kcal/mol]		
	NitTv1	NitAg	NitSh
Fumaronitrile (**1a**)	−0.15	−1.0	−0.77
3-Phenylpropionitrile (**2a**)	−3.49	− ^1^	n.i.
β-Cyano-L-alanine (**3a**)	−2.82	n.i.	−2.60
Benzonitrile (**4a**)	n.i.	n.i.	−4.56
2-Cyanopyridine (**5a**)	n.i.	n.i.	−4.22
4-Cyanopyridine (**6a**)	n.i.	n.i.	n.i.
(*R*)-Mandelonitrile (**7a**)	n.i.	n.i.	−5.18
(*S*)-Mandelonitrile (**8a**)	n.i.	n.i.	−4.78
Hydrogen cyanide (HCN)	n.i.	−3.50	−3.12

^1^ In this case, the substrate was docked in the active site close to C, but the nitrogen in the cyano group of the substrate is rotated outwards from the catalytic E and K, different from the proposed orientation of the intermediate in the 4hg5 crystal [30]. n.i. = not identified. In this case, the program Glide was unable to dock the substrate with the defined geometrical constraints (see text).

**Table 2:** Relative activities of the selected nitrilases.

Substrate	Relative Activity [%]		
	NitTv1	NitAg	NitSh
Fumaronitrile (**1a**)	100 ^1^	0.140 ± 0.003	0.199 ± 0.003
3-Phenylpropionitrile (**2a**)	6.26 ± 0.13	0	0
β-Cyano-L-alanine (**3a**)	7.96 ± 1.57	0	traces
Benzonitrile (**4a**)	0	0	0.135 ± 0.010
2-Cyanopyridine (**5a**)	traces	0	1.21 ± 0.03
4-Cyanopyridine (**6a**)	5.26 ± 0.26	traces	traces
HCN	0	100 ^2^	100 ^3^

^1^ Activity of 2.30 ± 0.06 U/mg dry cell weight was taken as 100%. ^2^ Activity of 3.22 ± 0.50 U/mg dry cell weight was taken as 100%. ^3^ Activity of 153 ± 20 U/mg dry cell weight was taken as 100%.

**Table 3:** Phylogenetic distribution of nitrilases in Basidiomycota.

Order ^1^ [Number of Sequences]	Genus ^1^ [Number of Sequences]		
	Clade 1	Clade 2	Others
Agaricales (38)	*Armillaria* (3) *Cylindrobasidium* (1) *Dendrothele* (1) *Fistulina* (1) *Gymnopus* (1) *Hypsizygus* (1) *Lentinus* (1) *Moniliophthora* (1) *Pleurotus* (1) *Pluteus* (1) *Schizophyllum* (1) *Termitomyces* (1)	*Armillaria* (5) *Dendrothele* (1) *Gymnopus* (1) *Lentinus* (1) *Moniliophthora* (3) *Mycena* (1) *Pleurotus* (2)	*Agaricus* (1) *Cylindrobasidium* (1) *Dendrothele* (3) *Pluteus* (1) *Moniliophthora* (2) *Mycena* (2)
Amylocorticiales (2)	*Plicaturopsis* (1)	*Plicaturopsis* (1)	
Atheliales (1)			*Fibulanrhizoctonia* (1)
Auriculariales (7)	*Auricularia* (1) *Exidia* (1)		*Auricularia* (4 ^2^) *Exidia* (1 ^3^)
Cystofilobasidiales (2)			*Xanthophylomyces* (2)
Boletales (7)	*Coniophora* (1) *Hydnomerulius* (1) *Paxillus* (1) *Rhizopogon* (2) *Serpula* (1) *Suillus* (1)		
Cantharellales (12)	*Botryobasidium* (1) *Rhizoctonia* (4)	*Rhizoctonia* (7)	
Corticiales (1)	*Punctularia* (1)		
Dacrymycetales (2)	*Calocera* (1) *Dacryopinax* (1)		
Geastrales (1)	*Sphaerobolus* (1)		
Gloeophyllales (2)			*Neolentinus* (1) *Heliocybe* (1)
Hymenochaetales (5)	*Fomitiporia* (1) *Phellinidium* (1) *Rickenella* (1) *Sanghuangporus* (1) *Schizopora* (1)		
Jaapiales (1)	*Jaapia* (1)		
Polyporales (16)	*Dichomitus* (1) *Ganoderma* (1) *Gelatoporia* (1) *Grifola* (1) *Obba* (1) *Phanerochaete* (3) *Phlebiopsis* (1) *Polyporus* (2) *Steccherinum* (1) *Trametes* (4)		
Russulales (10)	*Dentipellis* (1) *Heterobasidion* (1) *Peniophora* (1) *Stereum* (1)	*Bondarzewia* (1) *Dentipellis* (1) *Hericium* (2) *Heterobasidion* (1)	*Stereum* (1 ^3^)
Trechisporales (2)	*Sistotremastrum* (2)		
Tremellales (21)			*Cryptococcus* (4) *Kockovaella* (1) *Kwoniella* (13) *Naematelia* (2) *Saitozyma* (1)
Trichosporonales (4)			*Apiotrichum* (2) *Cutaneotrichosporon* (1) *Trichosporon* (1)

^1^ Assigned according to https://www.ncbi.nlm.nih.gov/Taxonomy. ^2^ three cyanide hydratases and an arylacetonitrilase. ^3^ cyanide hydratase. Note: Orders belonging to the class Tremellomycetes and Dacrymycetes are highlighted in light grey and dark grey, respectively. Other orders belong to class Agaricomycetes.

## Supplementary Materials

Supplementary materials can be found at https://www.mdpi.com/1422-0067/20/23/5990/s1.

## Abbreviations

ACN	Acetonitrile
BN	Benzonitrile
β-CA	β-Cyano-L-alanine
2CP	2-Cyanopyridine
4CP	4-Cyanopyridine
CynH	Cyanide hydratase
CynD	Cyanide dihydratase
DL	Desolvation line
DMSO-*d*_6_	Deuterated dimethylsulfoxide
ESI	Electrospray ionization
FN	Fumaronitrile
GDT	Global Distance Test
HB	Hydrogen bond
HGT	Horizontal gene transfer
IPTG	Isopropyl β-D-1-thiogalactopyranoside
MN	Mandelonitrile
MUSCLE	Multiple Sequence Comparison by Log-Expectation
NitAg	Nitrilase from *Armillaria gallica*
NitSh	Nitrilase from *Stereum hirsutum*
NitTv1	Nitrilase from *Trametes versicolor*
NLase	Nitrilase
PAN	Phenylacetonitrile
PPN	3-Phenylpropionitrile
RP	Reverse phase
