# Supplementary material for: Genetic and Functional Diversity of Nitrilases in Agaricomycotina
Source: Int J Mol Sci. 2019 Nov 28;20(23):5990. doi: 10.3390/ijms20235990 (PMC6928751; doi:10.3390/ijms20235990)
Supplement: Supplementary file 1 [file ijms-20-05990-s001.pdf]

Supplementary material – gene and protein sequences

Figure. S1 Sequences of genes optimized for expression in *Escherichia coli* and the corresponding nitrilases from *Trametes versicolor* (A), *Armillaria gallica* (B) and *Stereum hirsutum* (C) with restriction sites underlined.

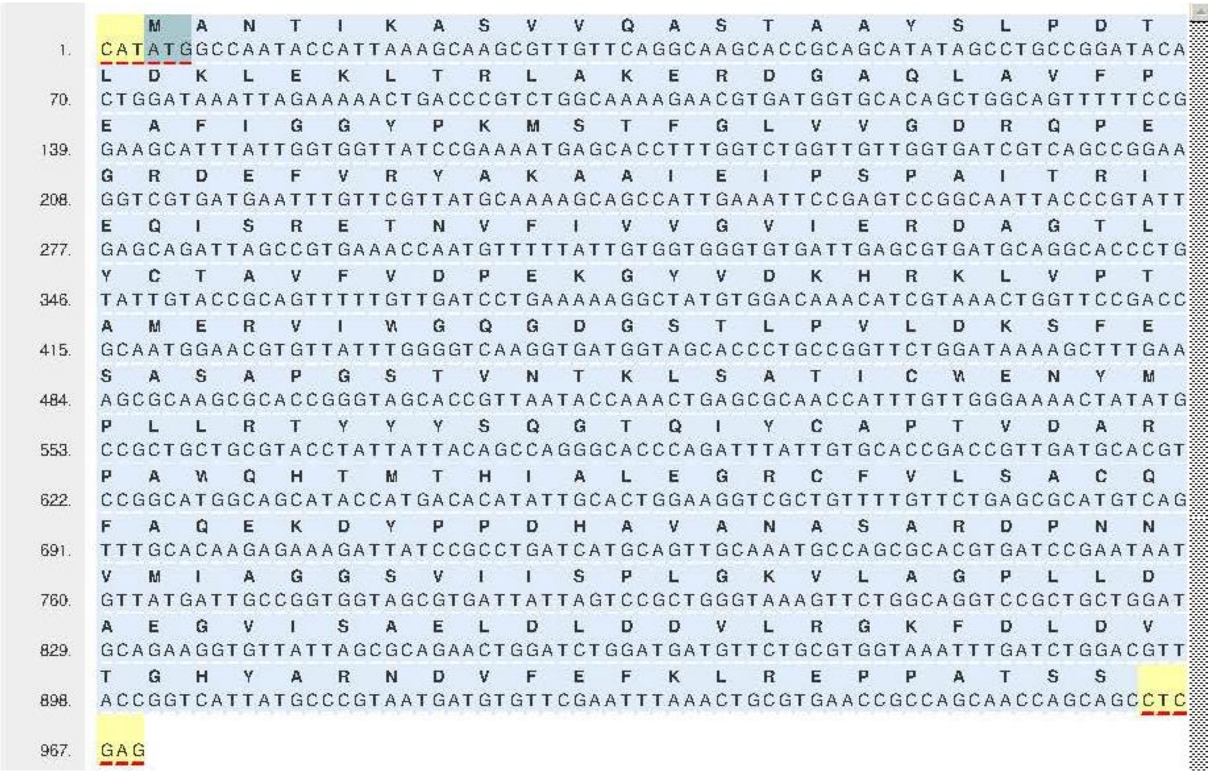

A

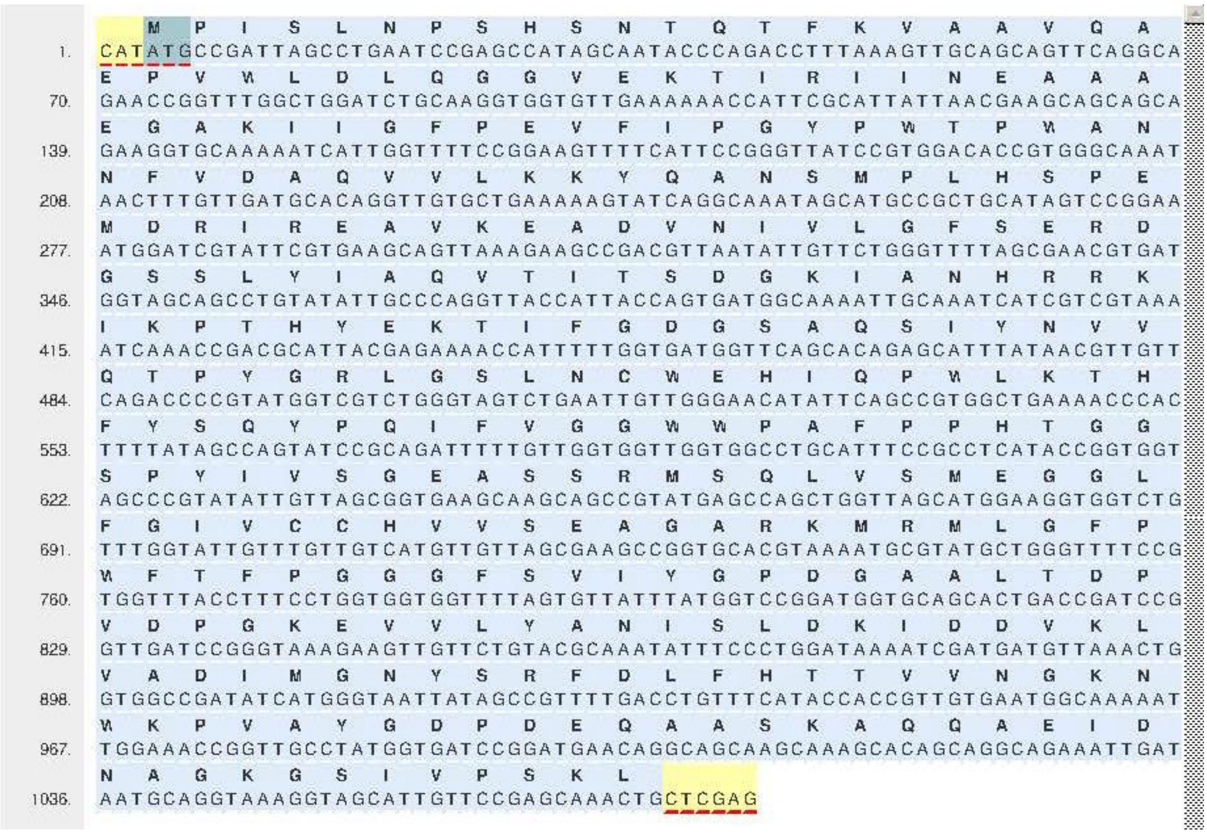

M P I S L N P S H S N T Q T F K V A A V Q A
1. CATATGCCGATTAGCCTGAATCCGAGCCATAGCAATACCCAGACCTTTAAAGTTGCAGCAGTTCAGGCA
E P V W L D L Q G G V E K T I R I I N E A A A
70. GAACCGGTTTGGCTGGATCTGCAAGGTGGTGTTGAAAAAACCATTCGCATTATTAACGAAGCAGCAGCA
E G A K I I G F P E V F I P G Y P W T P W A N
139. GAAGGTGCAAAAATCATTGGTTTTCCGGAAGTTTTCATTCCGGGTTATCCGTGGACACCGTGGGCAAAT
N F V D A Q V V L K K Y Q A N S M P L H S P E
208. AACTTTGTTGATGCACAGGTTGTGCTGAAAAAGTATCAGGCAAATAGCATGCCGCTGCATAGTCCGGAA
M D R I R E A V K E A D V N I V L G F S E R D
277. ATGGATCGTATTCGTGAAGCAGTTAAAGAAGCCGACGTTAATATTGTTCTGGGTTTTAGCGAACGTGAT
G S S L Y I A Q V T I T S D G K I A N H R R K
346. GGTAGCAGCCTGTATATTGCCCAGGTTACCATTACCAGTGATGGCAAAATTGCAAATCATCGTCGTAAA
I K P T H Y E K T I F G D G S A Q S I Y N V V
415. ATCAAACCGACGCATTACGAGAAAACCATTTTTGGTGATGGTTCAGCACAGAGCATTTATAACGTTGTT
Q T P Y G R L G S L N C W E H I Q P W L K T H
484. CAGACCCCGTATGGTCGTCTGGGTAGTCTGAATTGTTGGGAACATATTCAGCCGTGGCTGAAAACCCAC
F Y S Q Y P Q I F V G G W W P A F P P H T G G
553. TTTTATAGCCAGTATCCGCAGATTTTTGTTGGTGGTTGGTGGCCTGCATTTCCGCCTCATACCGGTGGT
S P Y I V S G E A S S R M S Q L V S M E G G L
622. AGCCCGTATATTGTTAGCGGTGAAGCAAGCAGCCGTATGAGCCAGCTGGTTAGCATGGAAGGTGGTCTG
F G I V C C H V V S E A G A R K M R M L G F P
691. TTTGGTATTGTTTGTTGTCATGTTGTTAGCGAAGCCGGTGCACGTAAAATGCGTATGCTGGGTTTTCCG
W F T F P G G G F S V I Y G P D G A A L T D P
760. TGGTTTACCTTTCCTGGTGGTGGTTTTAGTGTTATTTATGGTCCGGATGGTGCAGCACTGACCGATCCG
V D P G K E V V L Y A N I S L D K I D D V K L
829. GTTGATCCGGGTAAAGAAGTTGTTCTGTACGCAAATATTTCCCTGGATAAAATCGATGATGTTAAACTG
V A D I M G N Y S R F D L F H T T V V N G K N
898. GTGGCCGATATCATGGGTAATTATAGCCGTTTTGACCTGTTTCATACCACCGTTGTGAATGGCAAAAAT
W K P V A Y G D P D E Q A A S K A Q Q A E I D
967. TGGAAACCGGTTGCCTATGGTGATCCGGATGAACAGGCAGCAAGCAAAGCACAGCAGGCAGAAATTGAT
N A G K G S I V P S K L
1036. AATGCAGGTAAAGGTAGCATTGTTCCGAGCAAACTGCTCGAG

B

```
       M  P  I  T  Q  Y  K  A  A  A  V  T  S  E  P  C  W  F  D  L  E  A
1.     CATATGCCGATCACACAGTATAAAGCAGCAGCAGTTACCAGCGAACCGTGTTGGTTTGATCTGGAAGCC
       G  V  Q  K  T  I  S  F  I  N  E  A  G  Q  A  G  S  K  L  I  A  F  P
70.    GGTGTTCAGAAAACCATTAGCTTTATTAACGAAGCAGGTCAGGCAGGTAGCAAACTGATTGCATTTCCG
       E  V  W  I  P  G  Y  P  Y  W  M  W  K  V  T  Y  Q  Q  S  L  P  L  L
139.   GAAGTTTGGATTCCGGGTTATCCGTATTGGATGTGGAAAGTTACCTATCAGCAGAGCCTGCCGCTGCTG
       K  S  Y  R  E  N  S  L  P  V  D  S  E  E  M  R  R  I  R  R  A  A  R
208.   AAAAGCTATCGTGAAAATAGCCTGCCGGTTGATAGCGAAGAAATGCGTCGTATTCGTCGTGCAGCACGT
       D  N  H  I  Y  V  S  M  G  F  S  E  I  D  H  A  T  L  Y  L  S  Q  V
277.   GATAATCATATTTATGTTAGCATGGGCTTCAGCGAAATTGATCATGCAACCCTGTATCTGAGCCAGGTT
       L  I  S  P  T  G  D  V  L  N  H  R  R  K  I  K  P  T  H  V  E  K  L
346.   CTGATTAGCCCGACCGGTGATGTTCTGAATCATCGTCGTAAAATCAAACCGACGCATGTTGAAAAACTG
       V  Y  G  D  G  D  G  D  T  F  L  S  V  V  D  T  D  L  G  R  L  G  Q
415.   GTGTATGGTGACGGTGATGGTGATACCTTTCTGAGCGTTGTTGATACCGATCTGGGTCGTCTGGGTCAG
       L  N  C  W  E  N  M  N  P  F  L  K  S  L  N  I  A  M  G  E  Q  I  H
484.   CTGAATTGTTGGGAAAATATGAATCCGTTTCTGAAAAGCCTGAATATTGCAATGGGTGAGCAGATTCAT
       I  A  A  W  P  V  Y  P  G  K  E  T  L  K  Y  P  D  P  A  T  N  V  A
553.   ATCGCAGCATGGCCTGTTTATCCGGGTAAAGAAACCCTGAAATATCCGGATCCTGCAACCAATGTTGCA
       E  P  A  S  D  I  V  T  P  A  Y  A  L  E  T  A  T  W  T  L  A  P  F
622.   GAACCGGCAAGCGATATTGTTACCCCTGCCTATGCACTGGAAACCGCAACCTGGACACTGGCACCGTTT
       Q  R  L  S  V  E  G  L  K  K  N  T  P  A  G  M  E  P  E  T  D  P  S
691.   CAGCGTCTGAGCGTGGAAGGTCTGAAAAAAAACACACCGGCAGGTATGGAACCGGAAACCGATCCGAGC
       T  Y  N  G  H  A  R  I  Y  R  P  D  G  S  L  V  V  K  P  D  K  D  F
760.   ACCTATAATGGTCATGCACGTATTTATCGTCCGGATGGTAGCCTGGTTGTTAAACCGGATAAAGATTTT
       D  G  L  L  Y  V  D  I  D  L  N  E  S  H  L  T  K  A  L  G  D  F  A
829.   GATGGTCTGCTGTATGTGGATATCGATCTGAATGAAAGCCATCTGACCAAAGCACTGGGTGATTTTGCA
       S  G  H  Y  M  R  P  D  L  I  R  L  L  V  D  T  R  R  K  E  L  V  T
898.   AGCGGTCATTATATGCGTCCGGATCTGATTCGTCTGCTGGTTGATACCCGTCGTAAAGAACTGGTTACC
       E  A  D  P  D  G  G  V  A  T  Y  S  T  R  E  R  L  G  L  N  R  P  L
967.   GAAGCCGATCCGGATGGCGGTGTTGCAACCTATAGCACCCGTGAACGCCTGGGTCTGAATCGTCCGCTG
       D  P  P  K  D  E  R  H  G  I  V  G  V  A  G  Q  K  S  A  E  Q  R  K
1036.  GATCCGCCTAAAGATGAACGTCATGGTATTGTTGGTGTTGCAGGTCAGAAAAGCGCAGAACAGCGTAAA
       A  G  D  L
1105.  GCCGGTGATCTGCTCGAG
```

C

Supplementary data - Nitrilase sequences in *Agaricomycotina*

Table S1. List of nitrilase sequences in the species of *Agaricomycotina* according to GenBank and their classification into clades

Species	GenBank accession no.	Length (amino acids)	Clade	Closest characterized homologue Identity (%) /(Cover, %)
Agaricus bisporus v bisporus	XP_006462086.1	339		
Apiotrichum porosum	XP_028472322.1	352		
	XP_028475380.1	366		
Armillaria gallica	**PBL01211.1 (NitAg)**	**356**	**2**	**–**
	PBL01250.1	314	1	NitTv1 74 (96)
	PBK95426.1	336	2	NitAg 70 (93)
	PBK82882.1	355	2	NitAg 83 (96)
Armillaria ostoyae[a]	SJL05523.1	356	2	NitAg 97 (100)
	PBK74191.1	355	2	NitAg 84 (96)
	SJL05484.1	314		NitTv1 74 (97)
	PBK77460.1	314		NitTv1 73 (97)
Auricularia subglabra	**EJD42068.1 (NitAd)**[b]	**331**		**–**
	EJD55093.1	318	1	NitTv1 69 (96)
	EJD51184.1	365	CynH	NitSh 85 (97)
	EJD51215.1	346	CynH	NitSh 85 (100)
	EJD54336.1	365	CynH	NitSh 75 (94)
Bondarzewia mesenterica	THH19653.1			NitAg 56 (97)
Botryobasidium botryosum	KDQ08029.1	328	1	NitTv1 71 (92)
Calocera viscosa	KZO96291.1	310	1	NitTv1 55 (98)
Coniophora puteana	XP_007763492.1	339	1	NitTv1 69 (97)
Cryptococcus amylolentus	XP_018995183.1	342		
Cryptococcus neoformans	OXB39643.1	366		
	OWZ73108.1	309		

	XP_012046763.1	309		
Cutaneotrichosporon oleaginosum	XP_018275718.1	333		
Cylindrobasidium torrendii	KIY65145.1	311	1	NitTv1 67 (95)
	KIY71061.1	339		
Dacryopinax primogenitus	EJT97776.1	340	1	NitTv1 56 (91)
Dendrothele bispora	THV07691.1	316	1	NitTv1 74 (97)
	THV07653.1	367	2	NitAg 81 (94)
	THU83280.1	340		
	THU83281.1	349		
	THU93601.1	326		
Dentipellis fragilis	TFY69785.1	378	2	NitAg 68 (96)
	TFY69388.1	315	1	NitTv1 77 (97)
Dichomitus squalens	XP_007360414.1	319	1	NitTv1 87 (100)
Exidia glandulosa	KZW02628.1	313	1	NitTv1 70 (98)
	KZV92691.1	366	CynH	NitSh 82 (100)
Fibularhizoctonia sp.	KZP15294.1	371		
Fistulina hepatica	KIY50558.1	331	1	NitTv1 68 (96)
Fomitiporia mediterranea	XP_007265585.1	318	1	NitTv1 72 (97)
Ganoderma sinense	PIL31680.1	333	1	NitTv1 83 (98)
Gelatoporia subvermispora	EMD41986.1	317	1	NitTv1 77 (97)
Grifola frondosa	OBZ78624.1	295	1	NitTv1 76 (97)
Gymnopus luxurians	KIK70890.1	316	1	NitTv1 75 (96)
	KIK70855.1	305	2	NitAg 80 (85)
Heliocybe sulcata	TFK54302.1	322		
Hericium alpestre	TFY81337.1		2	NitAg 70 (96)
	TFY75083.1		2	NitAg 63 (91)
Heterobasidion irregulare	XP_009540410.1	315	1	NitTv1 74 (97)
	XP_009553498.1	353	2	NitAg 72 (96)
Hydnomerulius pinastri	KIJ68545.1	316	1	NitTv1 74 (96)
Hypsizygus marmoreus	RDB23399.1	313	1	NitTv1 73 (98)
Jappia argillacea	KDQ63493.1	342	1	NitTv1 64 (97)
Kockovaella imperatae	XP_021872624.1	332		
Kwoniella bestiolae	XP_019049898.1	346		
Kwoniella dejecticola	XP_018266860.1	344		
Kwoniella heveanensis	OCF37843.1	342		

	OCF30510.1	358		
	OCF41321.1	308		
	OCF35479.1	326		
Kwoniella magroviensis	XP_018999060.1	360		
	XP_018999159.1	351		
	OCF54328.1	376		
	OCF58370.1	360		
	XP_019000820.1	350		
	OCF62338.1	327		
Kwoniella pini	XP_019009856.1	345		
Lentinula edodes	GAW06039.1	360	2	NitAg 80 (97)
Lentinus tigrinus	RPD64846.1	320	1	NitTv1 85 (99)
Moniliophthora roreri	ESK97990.1	315	1	NitTv1 75 (96)
	ESK97956.1	358	2	NitAg 81 (97)
	ESK92265.1	330		
	ESK92248.1	379		
	KTB29936.1	330		
	KTB29977.1	330		
Mycena chlorophos	GAT44080.1	351	2	NitAg 72 (98)
	GAT47984.1	331		
	GAT55790.1	320		
Naematelia encephala	ORY27096.1	336		
	ORY35932.1	358		
Neolentinus lepideus	KZT26812.1	322		
Obba rivulosa	OCH94772.1	317	1	NitTv1 79 (97)
Paxillus involutus	KIJ21834.1	315	1	NitTv1 71 (96)
Peniophora sp.	KZV75469.1	308	1	NitTv1 71 (97)
Phanerochaete carnosa	XP_007390669.1	318	1	NitTv1 72 (96)
	XP_007401608.1	306	1	NitTv1 64 (97)
	XP_007391960.1	310	1	NitTv1 65 (98)
Phellinidium pouzarii	THH09479.1	339	1	NitTv1 65 (95)
Phlebiopsis gigantea	KIP12511.1	317	1	NitTv1 75 (96)
Pleurotus ostreatus	KDQ30886.1	322	1	NitTv1 74 (94)
	KDQ32741.1	304	2	NitAg 65 (85)
	KDQ30928.1	305	2	NitAg 76 (85)

Plicaturopsis crispa	KII93875.1	312	1	NitTv1 78 (97)
	KII92786.1	359	2	NitAg 68 (98)
Pluteus cervinus	TFK64162.1	334		
	TFK62976.1	323	1	NitTv1 71 (96)
Polyporus arcularius	TFK92769.1	321	1	NitTv1 84 (99)
Polyporus brumalis	RDX50451.1	321	1	NitTv1 84 (99)
Punctularia strigosozonata	XP_007379045.1	314	1	NitTv1 68 (95)
Rhizoctonia solani	CUA68841.1	320	1	NitTv1 65 (98)
	CEL62656.1	386	1	NitTv1 65 (98)
	EUC63334.1	318	1	NitTv1 66 (95)
	KDN41742.1	322	1	NitTv1 64 (92)
	EUC56910.1	329	2	NitAg 79 (89)
	KDN40720.1	364	2	NitAg 75 (96)
	CCO28557.1	363	2	NitAg 75 (96)
	CCO35235.1	316	2	NitAg 70 (99)
	CUA70852.1	352	2	NitAg 74 (96)
	ELU37431.1	331	2	NitAg 71 (94)
	KEP50135.1	364	2	NitAg 74 (96)
Rhizopogon vesiculosus	OJA16147.1	317	1	NitTv1 72 (98)
Rhizopogon vinicolor	OAX44198.1	317	1	NitTv1 72 (98)
Rickenella mellea	TDL28508.1	332	1	NitTv1 69 (95)
Saitozyma podzolica	RSH94106.1	387		
Sanghuangporus baumii	OCB88554.1	319	1	NitTv1 71 (95)
Schizophyllum commune	XP_003037202.1	317	1	NitTv1 71 (96)
Schizopora paradoxa	KLO14490.1	325	1	NitTv1 62 (97)
Serpula lacrymans	EGO05225.1	311	1	NitTv1 71 (97)
Sistotremastrum niveocremeum	KZS96174.1	311	1	NitTv1 70 (98)
Sistotremastrum suecicum	KZT44489.1	311	1	NitTv1 70 (98)
Sphaerobolus stellatus	KIJ45626.1	317	1	NitTv1 68 (97)
Steccherinum ochraceum	TCD67848.1	313		NitTv1 74 (99)
Stereum hirsutum	**XP_007307917.1 (NitSh)**	371	CynH	–
	XP_007298960.1	312	1	NitTv1 76 (96)
Suillus luteus	KIK46442.1	316	1	NitTv1 71 (98)
Termitomyces sp.	KNZ75542.1	314	1	NitTv1 69 (98)

Trametes cinnabarina	CDO73495.1	289	1	NitTv1 83 (81)
Trametes coccinea	OSC99476.1	338	1	NitTv1 91 (98)
Trametes pubescens	OJT10100.1	339	1	NitTv1 88 (100)
Trametes versicolor	**XP_008032838.1 (NitTv1)**	**320**	**1**	**–**
Trichosporon asahii	EKD01791.1	386		
Xanthophyllomyces dendrorhous	CDZ98326.1	319		
	CED82572.1	329		

[a]synonym: *Armillaria solidipes*

[b]NitAd from *Auricularia delicata*, re-classified *Auricularia subglabra*

[c]synonym: *Sanghuangporus baumii*

The enzymes overproduced in *E. coli* are marked in bold. NitAg, NitSh and NitTv1 were produced in this study, NitAd in the previous study (ref. [16] in the main manuscript). Proteins with ≥99% amino acid sequence identity, which occur in the same species, were discarded.

Sequences in blue do not belong to clade 1 or 2 or CynHs.

Supplementary data–Molecular modeling

Table S2. Normalized formation of HB during stable period of molecular docking (5–10 ns) in NitTv1 and NitAg nitrilase–fumaronitrile (FN) complexes.

NitTv1-FN	Interacting residues	K133-FN	K133-E46	K133-E140	C178-E46	V203-E46	V203-C178
	Formation of HB	0.92	0.965	0.915	0	0.78	0.9
NitAg-FN	Interacting residues	K137-FN	K137-E55	K137-E144	C172-E55	W197-E55	W197-C172
	Formation of HB	0.765	0.95	0.815	0.265	0	0.025
NitSh-FN	Interacting residues	K128-FN	K128-E46	K128-E135	C163-E46	P188-E46	P188-C163
	Formation of HB	0.71	0.91	0.77	0	0	0

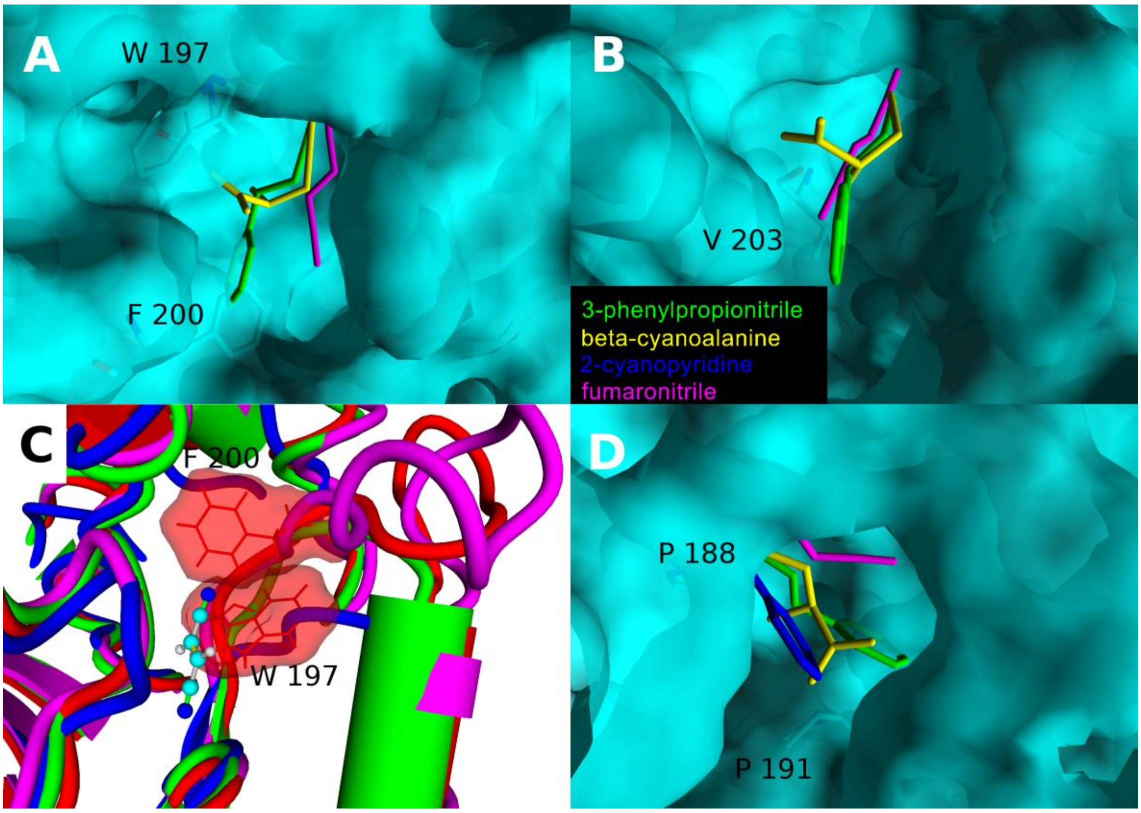

Figure. S2. Surface representation of the active sites of nitrilases with docked ligands. (A) NitAg with fumaronitrile (FN). The orientation of 3-phenylpropionitrile (3-PPN) and β-cyano alanine (β-CA) is made from structure alignment with NitTv1 complexed with corresponding ligands. (B) NitTv1 with FN, 3-PPN and β-CA. (C) Alignment of NitTv1 (blue), NitAg (red), NitSh (magenta) and 3wuy (green). W197 and F200 in NitAg are shown by red sticks, and the surface area occupied by them is marked in a red semitransparent colour. Part of the enzymes is hidden for clarity. (D) NitSh with FN, β-CA and 2-cyanopyridine, the orientation of which corresponds to complexes with NitSh. 3-PPN orientation corresponds to its position in NitTv1 complex.

Supplementary data–Product characterization

Products of fumaronitrile transformations by nitrilase NitTv1

To obtain samples for LC-MS, transformation of fumaronitrile (FN) was carried out using *Escherichia coli* whole cells carrying nitrilase (NLase) NitTv1 (dry cell weight 0.3 g/L) and 25 mM substrate in 50 mM Tris/HCl buffer, pH 8.0, with 150 mM NaCl (total volume 0.5 mL). The reaction proceeded at 30 °C and shaking (850 rpm) for 10 min. The reaction was terminated by adding 0.05 mL 2M HCl and the cells were removed by centrifugation. The supernatant was diluted with mobile phase (1:50) and analyzed by LC-MS (see Fig. S3 for the chromatogram and figure legend for *m/z* (ESI) data).

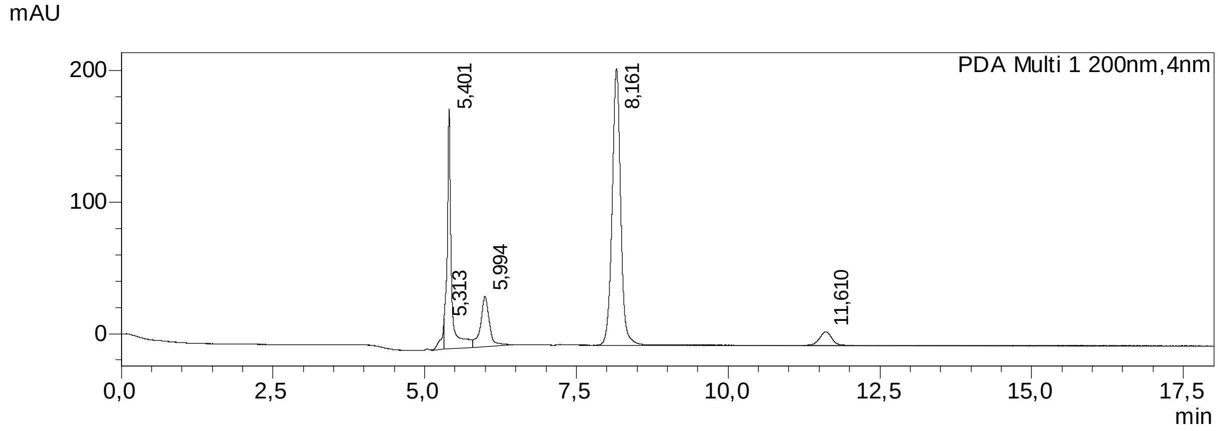

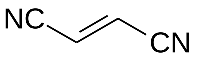

1a (RT=11.61 min)

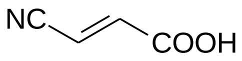

1b (RT=5.994 min)

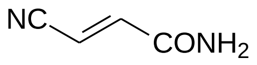

1c (RT=8.161 min)

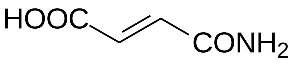

1d (RT=5.401 min)

Figure S3. HPLC of the products obtained from fumaronitrile (**1a**) using nitrilase NitTv1. Separations conditions: ACE 5 C8 (250 × 4 mm) column, mobile phase 10% acetonitrile in water, isocratic, flow rate 0.4 mL/min, 34 °C. *m/z* (ESI): **1b**: $[M-H]^-$ calculated for $C_4H_2NO_2$ 96.0, found 96; $[M+NO_3]^-$ calculated

for $C_4H_3N_2O_5$ 159.1, found 159; **1c**: $[M+HCOO]^-$ calculated for $C_5H_5N_2O_3$ 141.1, found 141; **1d**: $[M+Cl]^-$ calculated for $C_4H_5ClNO_3$ 150.0, found 150; The minor product with RT= 5.313 is fumaric acid: $[M+HCOO]^-$ calculated for $C_5H_5O_6$ 161.1, found 161.

To obtain products for NMR (Figs S4 and S5), the conditions of the transformation of FN were modified (dry cell weight 0.6 g/L, reaction time 60 min, total volume 50 mL). After removing the cells by centrifugation, the supernatant was extracted with ethylacetate at pH 8 (pH of the reaction mixture) and then at pH 2 (adjusted with 2M HCl). The organic fractions from each extraction were pooled, dried with Na_2SO_4 and filtered, and the solvent was removed at reduced pressure.

The product extracted at pH 8 contained **1c** as the major product (isolated yield 50%; 61 mg).

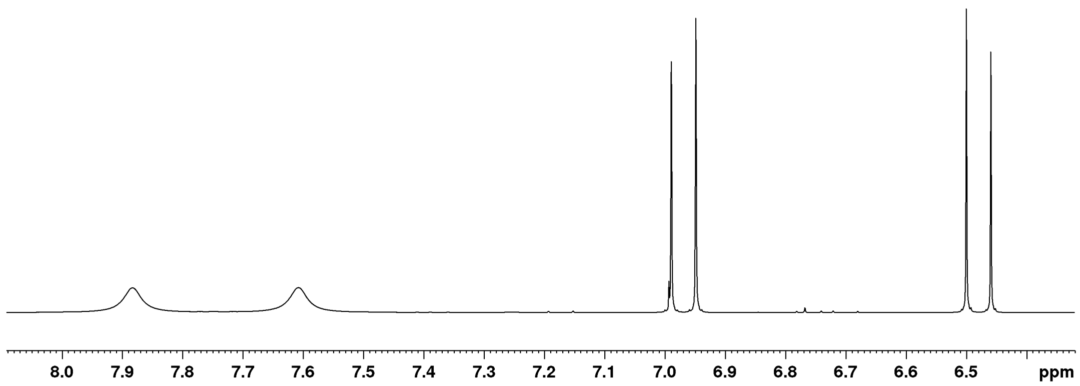

Figure S4. The detail of ^{1}H NMR spectrum of the product extracted at pH 8 (399.87 MHz, DMSO, 30 °C) with major compound **1c**

NMR data: **1c** - ^{1}H NMR (399.87 MHz, DMSO, 30 °C): 6.479 (1H, d, *J* = 16.3 Hz, CH), 6.969 (1H, d, *J* = 16.3 Hz, CH), 7.608 (1H, br s, NH_2-u), 7.883 (1H, br s, NH_2-u); ^{13}C NMR (100.55 MHz, DMSO, 30 °C): 108.65 (CH), 117.07 (CN), 144.05 (CH), 163.20 (CO).

The product extracted at pH 2.5 (isolated yield 17%; 20 mg) contained **1b** as the major product (62% of total product) .Compounds **1c**, **1d**, fumaric acid and its diamide were minor products with ca. 10%, 16%, 2% and 10% of the total product.

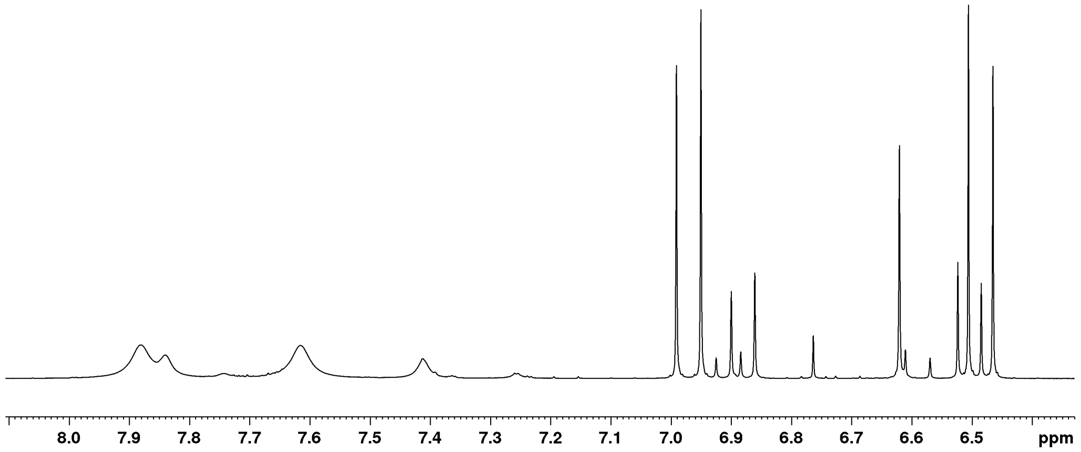

Figure S5. The detail of ^{1}H NMR spectrum of the product extracted at pH 2.5 (399.87 MHz, DMSO, 30 °C) with major compound **1b**

NMR data: **1b** - ^{1}H NMR (399.87 MHz, DMSO, 30 °C): 6.590 (1H, d, *J* = 16.4 Hz, CH), 6.904 (1H, d, *J* = 16.4 Hz, CH), OH not detected; ^{13}C NMR (100.55 MHz, DMSO, 30 °C): 111.66 (CH), 116.62 (CN), 142.77 (CH), 164.81 (CO); **1d** - ^{1}H NMR (399.87 MHz, DMSO, 30 °C): 6.504 (1H, d, *J* = 15.6 Hz, CH), 6.880 (1H, d, *J* = 15.6 Hz, CH), NH_2 not extracted; ^{13}C NMR (100.55 MHz, DMSO, 30 °C): 129.95 (CH), 137.20 (CH), 164.81 (CO), 166.46 (CO).

Products of fumaronitrile transformations by nitrilase NitAg

To obtain samples for LC-MS from the reaction using whole cells carrying NLase NitAg, transformation of FN was carried out analogously as described for NitTv1 above but with modifications (dry cell weight 4.5 g/L, reaction time 120 min, total volume 0.5 ml). The supernatant was diluted with mobile phase (1:50) and analyzed by LC-MS (see Fig. S6 for the chromatogram and figure legend for *m/z* (ESI) data).

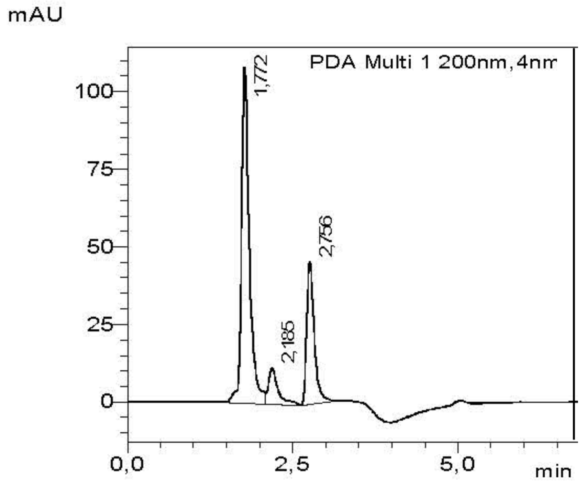

Figure S6. HPLC of the products obtained from fumaronitrile (**1a**, RT = 2.756) using nitrilase NitAg. Separation conditions: Chromolith RP 18e (100 × 3 mm) column, mobile phase 10% acetonitrile in water, isocratic, flow rate 0.4 mL/min, 34 °C. *m/z* (ESI): **1b**: $[M-H]^-$ calculated for $C_4H_2NO_2$ 96.0, found 96; **1c**: $[M+Cl]^-$ $]^-$ calculated for $C_4H_3N_2OCl$ 131.1, found 131. See Fig. S3 for the product structures.

Products of fumaronitrile transformations by nitrilase NitSh

Transformation of FN by NitSh was carried out analogously as described for NitTv1 above but with modifications (dry cell weight 3 g/L, reaction time 60 min and total

volume 50 mL). The products were isolated in the same way as in the previous experiment. The product extracted at pH 8 (27 mg) contained a mixture of the residual substrate **1a** and product **1c** at a ratio of ca. 2 : 3. The product extracted at pH 2.5 contained compound **1b** as the major product (isolated yield 69%; 41 mg). The products were analyzed by NMR (Figs S7 and S8).

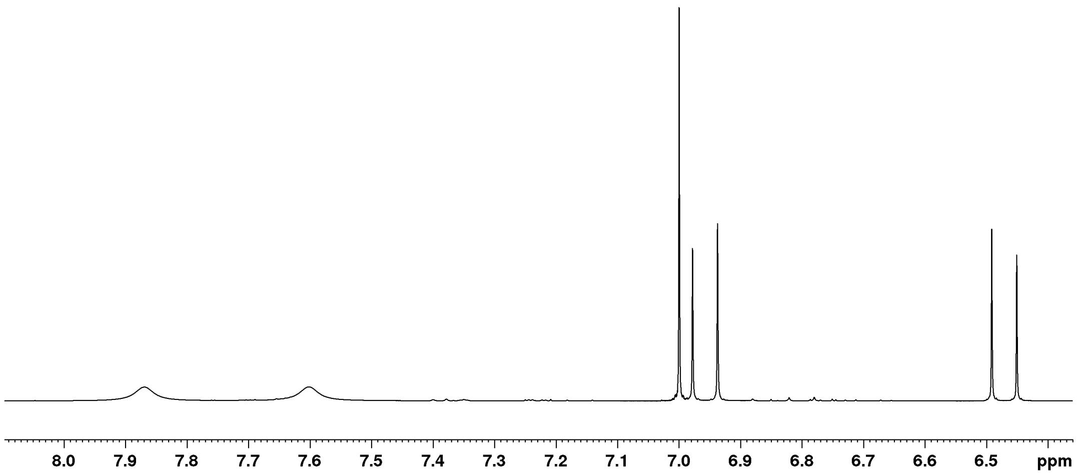

Figure S7. The detail of ^{1}H NMR spectrum of the product extracted at pH 8 (399.87 MHz, DMSO, 30 °C)

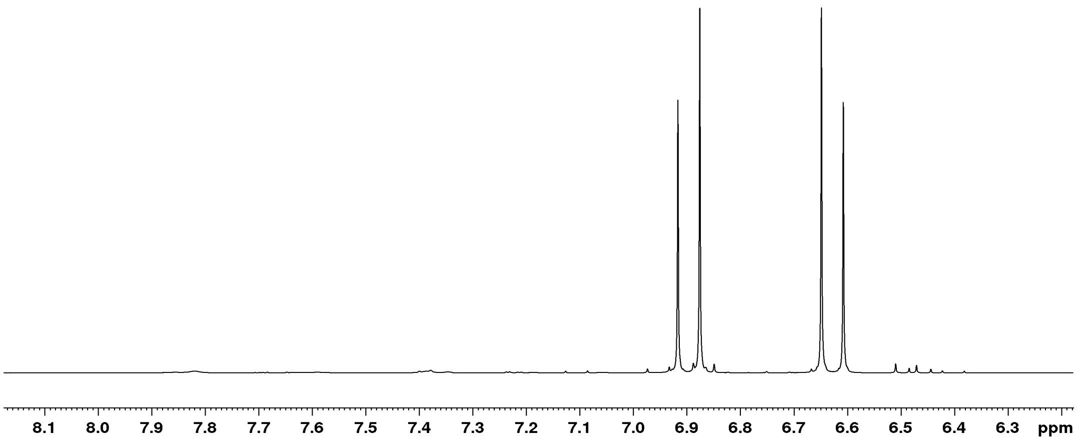

Figure S8. The detail of ^{1}H NMR spectrum of the product extracted at pH 2.5 (399.87 MHz, DMSO, 30 °C)

Product of 3-phenylpropionitrile transformation by nitrilase NitTv1

The transformation of 3-phenylpropionitrile (PPN) by NitTv1 was carried out analogously as described for FN above but with modifications (dry cell weight 3 g/L, reaction time 120 min, total volume 0.5 mL). The sample for LC-MS was prepared as described for FN above (see Fig. S9 for the chromatogram and figure legend for *m/z* (ESI) data).

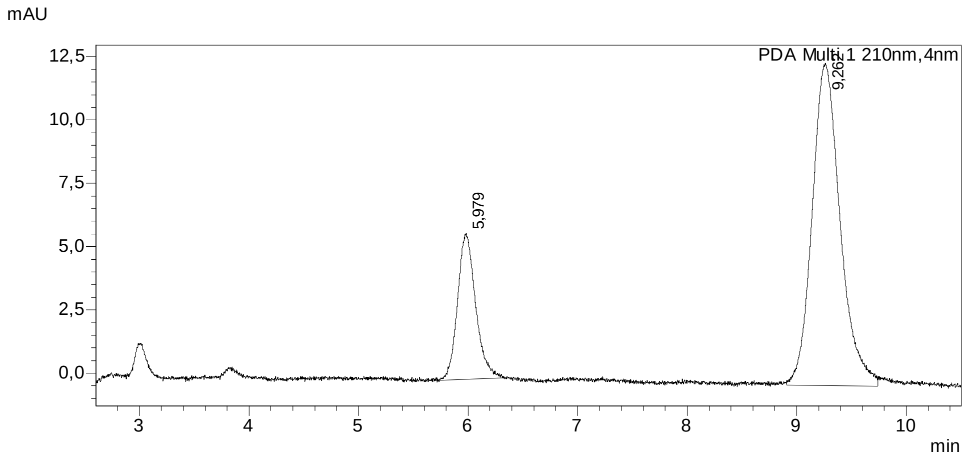

Figure S9. HPLC of the products obtained from 3-phenylpropionitrile (RT = 9.262 min) using nitrilase NitAg. Separation conditions: Chromolith RP 18e (100 × 3 mm) column, mobile phase 10% acetonitrile in water, isocratic, flow rate 0.4 mL/min, 34 °C.

m/z (ESI): 3-phenylpropionic acid (RT = 5.979 min): $[M\text{-}H\text{+}CH_3OH]^-$ calculated. for $C_{10}H_{13}O_3$ 181.2, found 181.

Products of β-cyanoalanine transformation by nitrilase NitTv1

The transformation of β-cyanoalanine (β-CA) by NLase NitTv1 was carried out analogously as described for FN but with modifications (dry cell weight 0.6 g/L,

reaction time 60 min, total volume 50 mL). The supernatant was lyophilized and analyzed by NMR (Figs S10 and S11).

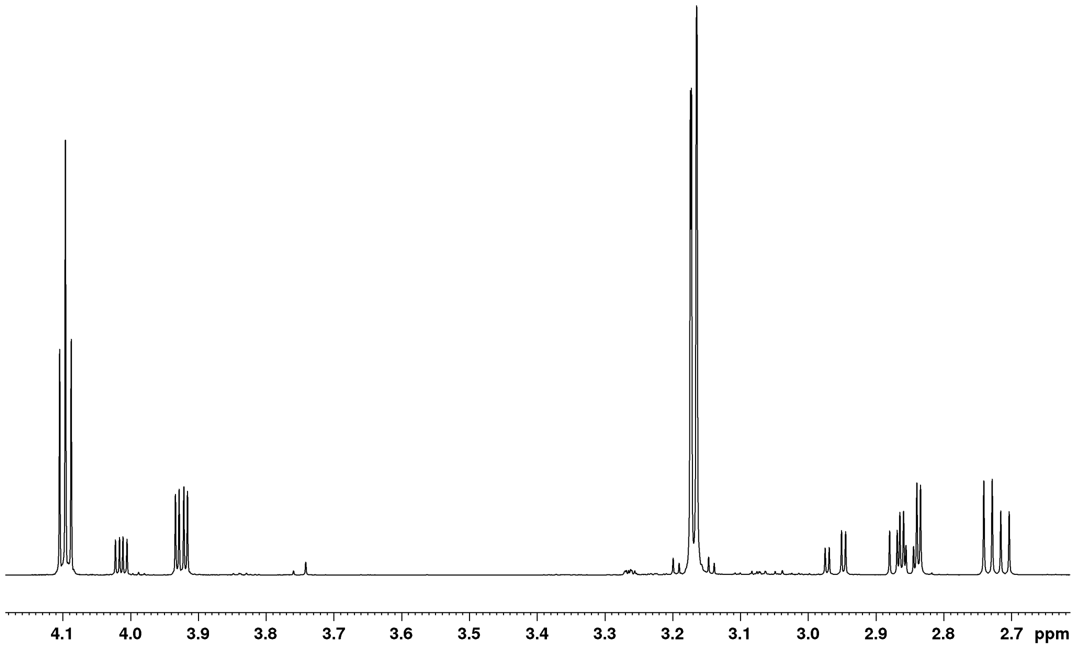

Figure S10. The detail of ^{1}H NMR spectrum of the product extracted at pH 8 (700.13 MHz, D_2O, 30 °C). Approximate ratio Asp: Asn = 71: 29. This sample also contained a significant amount of the residual substrate.

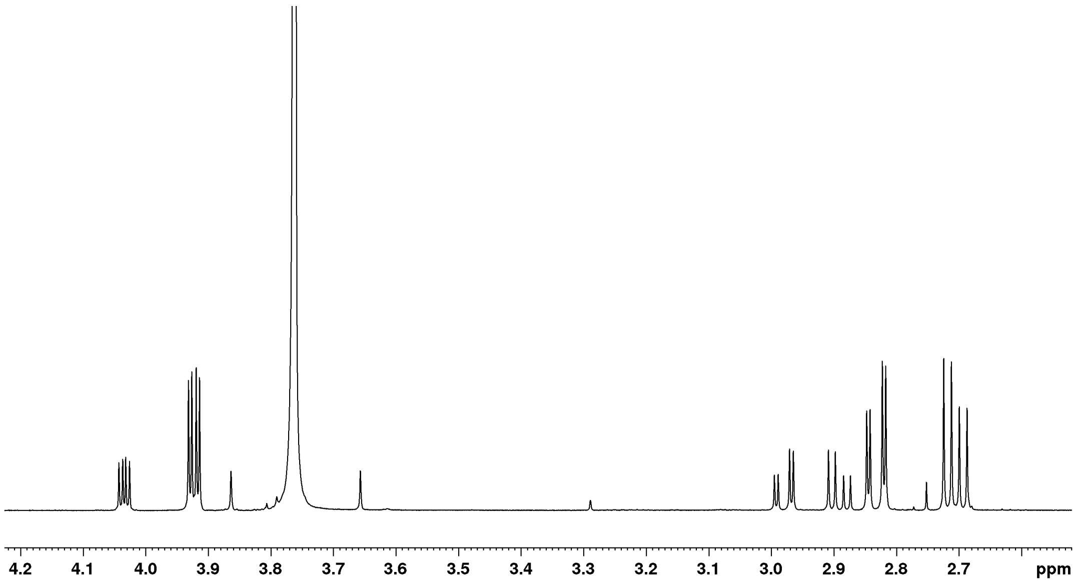

Figure S11. The detail of ^{1}H NMR spectrum of the product extracted at pH 2.5 (700.13 MHz, D_2O, 30 °C). Approximate ratio Asp: Asn = 73:27.

<u>NMR data</u>: Aspartic acid - ^{1}H NMR (700.13 MHz, D_2O, 30 °C): 2.705 (1H, dd, *J* = 17.5, 8.7 Hz, H-βu), 2.832 (1H, dd, *J* = 17.5, 3.8 Hz, H-βd), 3.923 (1H, dd, *J* = 8.7, 3.8 Hz, H-α); ^{13}C NMR (176.05 MHz, D_2O, 30 °C): 36.76 (C-β), 52.46 (C- α), 174.43 (CO), 177.79 (CO); Asparagine - ^{1}H NMR (700.13 MHz, D_2O, 30 °C): 2.891 (1H, dd, *J* = 17.0, 7.6 Hz, H-βu), 2.980 (1H, dd, *J* = 17.0, 4.3 Hz, H-βd), 4.034 (1H, dd, *J* = 7.6, 4.3 Hz, H-α); ^{13}C NMR (176.05 MHz, D_2O, 30 °C): 34.72 (C-β), 51.52 (C- α), 173.49 (CO), 174.66 (CO)

Products of 2-cyanopyridine transformation by nitrilase NitSh

The transformation of 2-cyanopyridine (2CP) by NitSh was carried out analogously as described for FN above but with modifications (dry cell weight 0.3 g/L, reaction time 10 min, total volume 0.5 ml). The samples for LC-MS were prepared as described for FN above (see Fig. S12 for the chromatogram and figure legend for *m/z* (ESI) data).

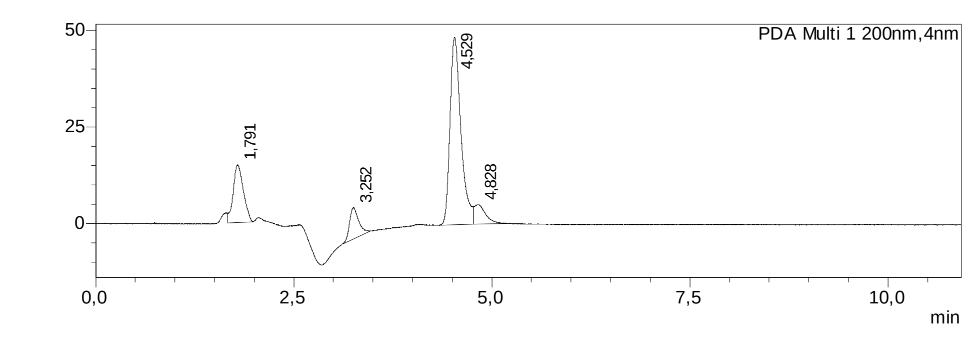

Figure S12 HPLC of the products of biotransformation of 2-cyanopyridine by nitrilase NitSh. Separation conditions: Chromolith RP 18e (100 × 3 mm) column, mobile phase 10% acetonitrile in water, isocratic, flow rate 0.4 mL/min, 34 °C. *m*/*z* (ESI): picolinic acid (RT= 1.791): $[M\text{-}H]^-$ calculated for $C_6H_4NO_2$ 122.1, found 122; $[M\text{-}H\text{+}H_2O]^-$ calculated for $C_6H_6NO_3$ 140.0, found 140; picolinamide (RT= 3.252): $[M\text{+}Na\text{+}CH_3CN]^+$ calculated for $C_8H_9N_3NaO$ 186.1, found 186; 2-cyanopyridine, RT= 4.529.

Products of 4-cyanopyridine transformation by nitrilase NitTv1

The transformation of 4-cyanopyridine (4CP) by NitTv1 was carried out analogously as described for FN above but with modifications (dry cell weight 3 g/L, reaction time 120 min). The samples for LC-MS were prepared as described for FN above (see Fig. S13 for the chromatogram and figure legend for *m*/*z* (ESI) data).

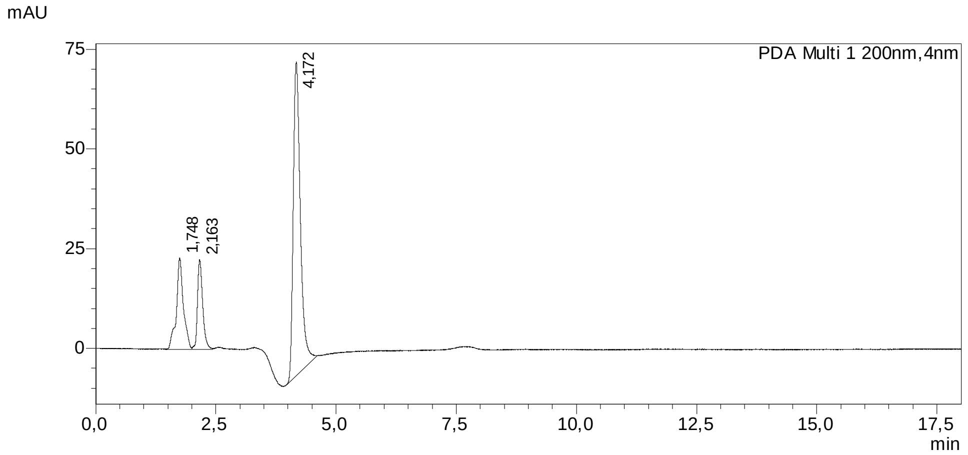

Figure S13 HPLC of the products of biotransformation of 4-cyanopyridine by nitrilase NitTv1. Separation conditions: Chromolith RP 18e (100 × 3 mm) column, mobile phase 10% acetonitrile in water, isocratic, flow rate 0.4 mL/min, 34 °C. *m*/*z*

(ESI): isonicotinic acid (RT= 1.748): $[M-H]^-$ calculated for $C_6H_4NO_2$ 122.1, found 122; $[M-H+H_2O]^-$ calculated for $C_6H_6NO_3$ 140.0, found 140; isonicotinamide (RT= 2.162): $[M+Cl]^-$ calculated for $C_6H_5N_2OCl$ 157.1, found 157; 4-cyanopyridine, RT= 4.171.

Products of benzonitrile transformation by nitrilase NitSh

The transformation of benzonitrile (BN) by NitSh was carried out as described for FN above but with modifications (dry cell weight 0.3 g/L, reaction time of 5 min, total volume 0.5 ml). The product (benzoic acid) was determined by HPLC as described in Materials and methods and its UV spectrum was compared with that of the authentic standards (absorption maximum at 228.7). No significant amount of benzamide (absorption maximum at 225.2 nm) was found in the reaction mixture
